# Supplementary material for: Unraveling the role of vaporization momentum in self-jumping dynamics of freezing supercooled droplets at reduced pressures
Source: Nat Commun. 2024 Feb 21;15:1567. doi: 10.1038/s41467-024-45928-2 (PMC10879204; doi:10.1038/s41467-024-45928-2)
Supplement: Supplementary file 1 — Supplementary Information [file 41467_2024_45928_MOESM1_ESM.pdf]

Supplementary Information for

Unraveling the Role of Vaporization Momentum in Self-Jumping Dynamics of Freezing Supercooled Droplets at Reduced Pressures

Xiao Yan[1,2,3=]*, Samuel C. Y. Au[1=], Sui Cheong Chan[1], Ying Lung Chan[1], Ngai Chun Leung[1], Wa Yat Wu[1], Dixon T. Sin[1], Guanlei Zhao[4], Casper H. Y. Chung[1], Mei Mei[1], Yinchuang Yang[1], Huihe Qiu[1], Shuhuai Yao[1,5]*

[1]*Department of Mechanical and Aerospace Engineering, Hong Kong University of Science and Technology, Hong Kong, China*

[2]*Key Laboratory of Low-grade Energy Utilization Technologies and Systems, Chongqing University, Ministry of Education, Chongqing 400030, P.R. China*

[3]*Institute of Engineering Thermophysics, Chongqing University, Chongqing 400030, P.R. China*

[4]*State Key Laboratory of Automotive Safety and Energy, School of Vehicle and Mobility, Tsinghua University, Beijing 100084, China*

[5]*HKUST Shenzhen-Hong Kong Collaborative Innovation Research Institute, Futian, Shenzhen, China*

[=] *Equal contribution*

* *Corresponding author emails*: yanx23@cqu.edu.cn (Xiao Yan); meshyao@ust.hk (Shuhuai Yao)

Table of Contents

Supplementary Methods

Section S1: Depressurization processes

Section S2: Fabrication and characterization of the substrates

Supplementary Notes

Section S3: Calculation of the vaporization flux

Section S4: Characterization of intensive vaporization during recalescence

Section S5: Characterization of recalescence

Section S6: Vaporization-induced droplet sliding

Section S7: Characterization of droplet jumping

Section S8: Modeling of freezing droplet jumping

Section S9: Characterization of droplet deformation

Section S10: Overpressure analysis

Section S11: A qualitative analysis of time scales

Section S12: Effects of substrate adhesion on jumping dynamics

Section S13: Development of the regime map

Supplementary References

Supplementary Methods

Section S1. Depressurization Processes

Supplementary Fig. 1a shows the vacuum chamber for the droplet freezing tests. Upon vacuuming the chamber, the chamber pressure (measured by MKS® 627H Manometer) decreases to ~100 Pa within 15-20 s, after which the droplet starts to freeze (Supplementary Fig. 1b). Due to the reduced pressure, the droplet evaporates to shrink in volume and radius (Supplementary Fig. 1c). The evaporation mass flux J_{w} over the free surface of the unfrozen droplet can be estimated by:[1]

$$J_{\mathrm{w}} = \frac{1}{A_{\mathrm{d}}}\frac{dm}{dt}, \tag{1}$$

where h_{fg} is the liquid-to-gas latent heat of water, A_{d} is the surface area of the droplet, $\mathrm{d}m/dt$ is the mass (m) change rate of the evaporating droplet. The corresponding evaporative heat flux is $q''_{\mathrm{e}} = J_{\mathrm{w}} h_{\mathrm{fg}}$. Supplementary Fig. 1d shows J_{w} and q''_{w} for a droplet having an initial volume of 10 μL.

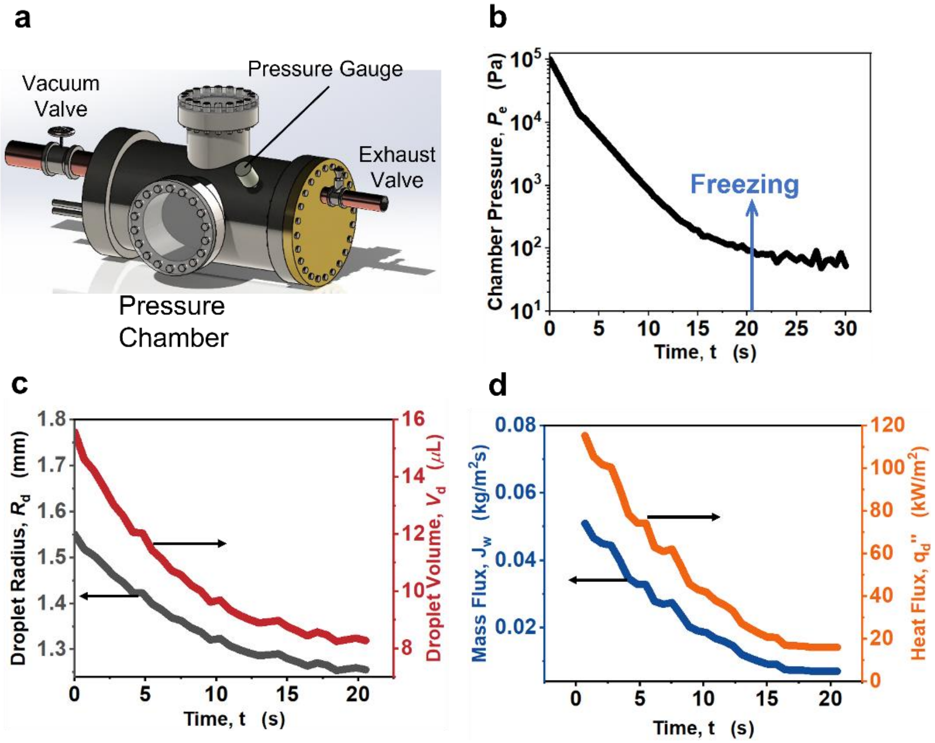

Supplementary Fig. 1. Low-pressure environment for droplet vaporization experiments. (a) Schematic of the chamber. (b) Pressure (absolute pressure) within the chamber during depressurization with

the droplet freezing moment indicated by the blue arrow. (c) Droplet volume as a function of time during depressurization upon freezing. (d) Mass flux J_{w} and heat flux q''_{d} as a function of time during depressurization upon freezing.

To characterize the thermodynamic conditions during depressurization, we measured the droplet temperature as a function of chamber pressure and presented the temperature-pressure curve against the water phase diagram (Supplementary Fig. 2c). The droplet temperature was measured by inserting a T-type fine (probe diameter of 0.6 mm) thermocouple into a droplet (with an initial volume of 15 μL) residing on the substrate (Supplementary Fig. 2b). The thermocouple measures the internal temperature within the droplet, which is higher than that at the droplet's free surface due to the evaporative cooling-induced temperature gradient. We note although the temperature contrast of the droplet's surface can be captured by the infrared (mid-IR, 1.5-5 μm) camera (FLIR SC7700) through the sapphire window (Supplementary Fig. 2c), the complex calibration to exclude the thermal radiation from the environment and reflection makes the IR imaging inefficient for precise temperature measurement of the icing droplet within the chamber. Thus, the IR imaging was only used to observe the ice nucleation and ice propagation as we did in Figure 2c in the manuscript. To estimate the droplet surface temperature, we assume a spherical droplet with a heat flux uniformly distributed over the droplet surface. The droplet surface temperature can be solved by Eq. S2 with known internal temperature (assuming at the droplet center) and mass flux (Supplementary Eq. 1):

$$T_{\mathrm{i}} = T_{\mathrm{o}} - q''_{\mathrm{e}} A_{\mathrm{d}} R_{\mathrm{t}}, \tag{2}$$

where R_{t} is the approximated thermal resistance between the droplet and thermocouple which is defined in Supplementary Eq. 3 below, where r_2 is the droplet radius, r_1 is the thermocouple probe radius, and k_{w} is the thermal conductivity of water:

$$R_{\mathrm{t}} = \frac{r_2 - r_1}{4\pi r_2 r_1 k_{\mathrm{w}}}. \tag{3}$$

The calculation shows that the droplet surface temperature immediately before the ice nucleation moment is -27.2°C, which is lower than the internal temperature (-12.0±0.5°C). The calculation is consistent with the IR imaging where a surface temperature of around -21°C was captured regardless of the effects of ambient thermal radiation.[2,3]

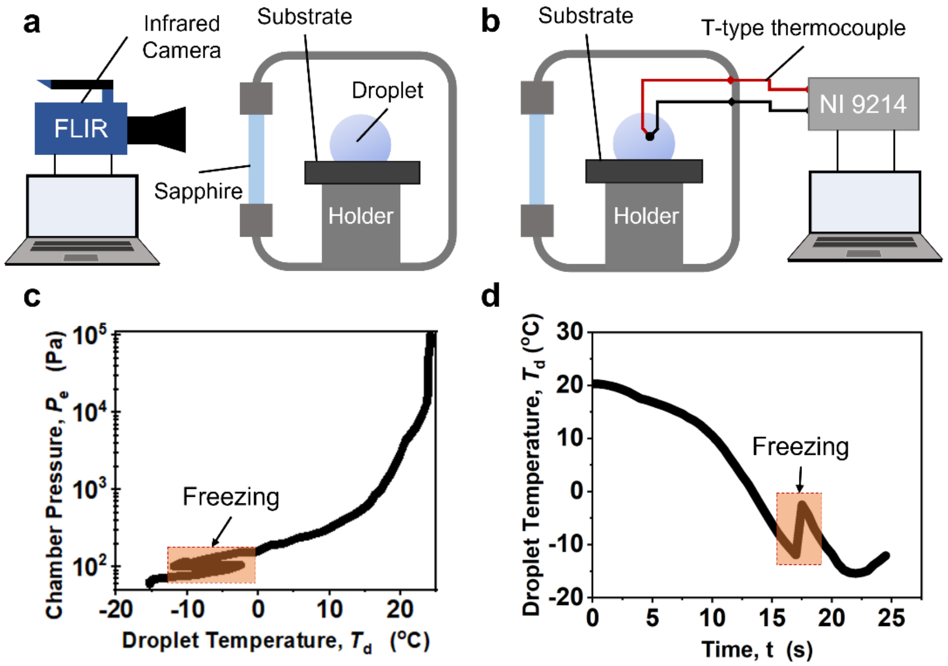

Supplementary Fig. 2. Temperature measurement. Experimental setup for droplet temperature measurement by (a) infrared thermal camera (FLIR SC 7700) and (b) T-type thermocouple. (c) Droplet temperature during chamber depressurization measured by a T-type thermocouple as a function of time, where the droplet nucleation happened at ~ 17 s with an instantaneous temperature jump. Time zero represents the moment when the chamber pressure starts to decrease. (d) The droplet temperature measured by the thermocouple plotted against chamber pressure. Droplet freezing occurred at ~ 100 Pa, along with an isobaric increase of droplet temperature from around -12°C to 0°C.

Section S2. Fabrication and Characterization of the Substrates

Three different surface samples were used in our experiments, namely S1, S2, and S3 (Supplementary Fig. 3). The samples were prepared by ablating the clean aluminum plates (99.5% in purity) with a linearly polarized picosecond laser (Edgewave PX series) with a wavelength of 1064 nm, pulse repetition rate of 1 MHz, and pulse width of 12 ps.[4,5] The average output power was 1.2 W. A galvanometer (Scanlab, Intellicube 14) with two mirrors and an F-Theta objective lens was installed to steer the laser beam and scan on the x-y plane of the aluminum plate in a 10 x 10 mm area. The intensity profile of the focused beam is Gaussian distributed and the half-width of the intensity profile is approximately 17.5 μm. The laser scans repeatedly a square grid path on surfaces S1 and S3, while parallel scanning is done on surface S2, leading to the construction of pillars or grooves. The scanning speed of the laser is maintained at 1 m/s, with 94.3% of scanning overlapping rate, where the spacing of adjacent later scanning lines can be adjusted. After laser processing, the laser-textured samples were cleaned sonication bath in acetone, isopropyl alcohol (IPA), and deionized (DI) water for 5 min each. The samples were then placed into a hot water bath of 90°C for 1 hour to form boehmite nanostructures. After drying under nitrogen gas, the samples were modified with heptadecafluorodecyltrimethoxy silane (TCI America, CAS #: 83048-65-1) via vapor phase deposition. Specifically, the samples were placed in a container with a vial of 1mL of silane-toluene solution (5% v/v). A lid was placed on top to seal the container, followed by heating in an atmospheric pressure oven at 90±5°C for 3 h. This process allowed for the adsorption of a highly conformal coating on the surfaces.[6] Supplementary Fig. 3 shows the SEM along with the contact angle of laser processed samples. All surfaces have an apparent static contact angle greater than 160° with a contact angle hysteresis less than 5°.[5] The solid fraction of the microstructure can be estimated by dividing the actual contact area A_{c} to the projected area A_{p}. For surfaces S1 and S3, we consider the microstructure similar to a frustum, where the contact area is the tip area of the frustum. We measured the tip radius r_{tip}=21.22 μm from Supplementary Fig. 3. Since the pitch between the frustum structure is p_{f}=100 μm and the frustums are squarely patterned on the surface, we estimate the solid fraction by using a unit surface consisting of a frustum in a square with sides equal to the pitch p_{f}=100 μm. Therefore, the actual contact area is $A_{\mathrm{C}} = \pi r_{\mathrm{tip}}^2$ and the projected area is $A_{\mathrm{p}} = p_{\mathrm{f}}^2$, which gives the solid fraction $\varphi_{\mathrm{m}} = A_{\mathrm{C}}/A_{\mathrm{p}} \approx 0.223$. For surface S2, it is a grooved surface where the solid fraction can be calculated by dividing the

width of the protruded surface w=44.7 μm by the pitch of the groove p_{g}=100 μm, resulting in a solid fraction $\varphi_{\mathrm{m}} = w/p_{\mathrm{g}} \approx 0.447$.

To obtain the solid fraction of nanostructure, we fabricated an aluminum (Al) surface only with nanostructures by placing a clean aluminum plate into a hot water bath at 90°C to grow the boehmite nanostructures. After the hot water bath, the nanostructured aluminum plate was dried under nitrogen gas and dipped into 0.5wt% silane – hexane solution for 1 hour and dried at 120°C in a furnace for 2 hours.[6] The solid fraction with respect to nanostructures can be solved for by the Cassie-Baxter equation:[7]

$$\cos\theta_{\mathrm{s}}^{\mathrm{app}} = -1 + \varphi_{\mathrm{n}}(\cos\theta_0 + 1), \quad (4)$$

where $\theta_{\mathrm{s}}^{\mathrm{app}}$ is the apparent static contact angle on the nanostructured aluminum surface and θ_0 is the intrinsic contact angle of the smooth aluminum surface coated with the hydrophobic silane.[6] For $\theta_{\mathrm{s}}^{\mathrm{app}} = 158^{\mathrm{o}}$ and $\theta_0 = 109^{\mathrm{o}}$, φ_{n} is estimated to be 0.108.[4]

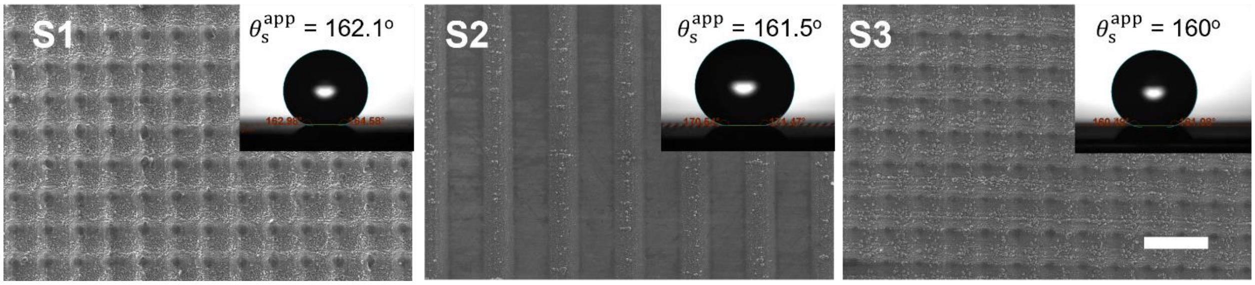

Supplementary Fig. 3. SEM micrographs and static contact angles (top right insets) of the laser-textured aluminum samples S1, S2, and S3. Apparent static contact angles were measured by a goniometer (Biolin Theta) using 6 μL deionized water droplets. Scale bar: 200 μm.

To confirm the self-jumping dynamics, we conducted additional experiments using a greater variety of surfaces having differing surface structures (Supplementary Fig. 4 and Table 1). The length scales of surface structures span a wider range from nanoscale (~100 nm) and microscale (~10 μm to ~100 μm) to macroscale (~ mm). The morphology of the structures varies from randomly distributed nanoblades, micropillars, microgrooves, re-entrant microgrids, microhills with nanowires, microlattice, to macrogrooves. All surfaces have nanostructures except the re-

entrant surfaces. See our previous studies for more details on the fabrication and characterization of the superhydrophobic Al surfaces,[8] CuO nanowire surfaces,[9,10] and re-entrant Si surfaces.[11]

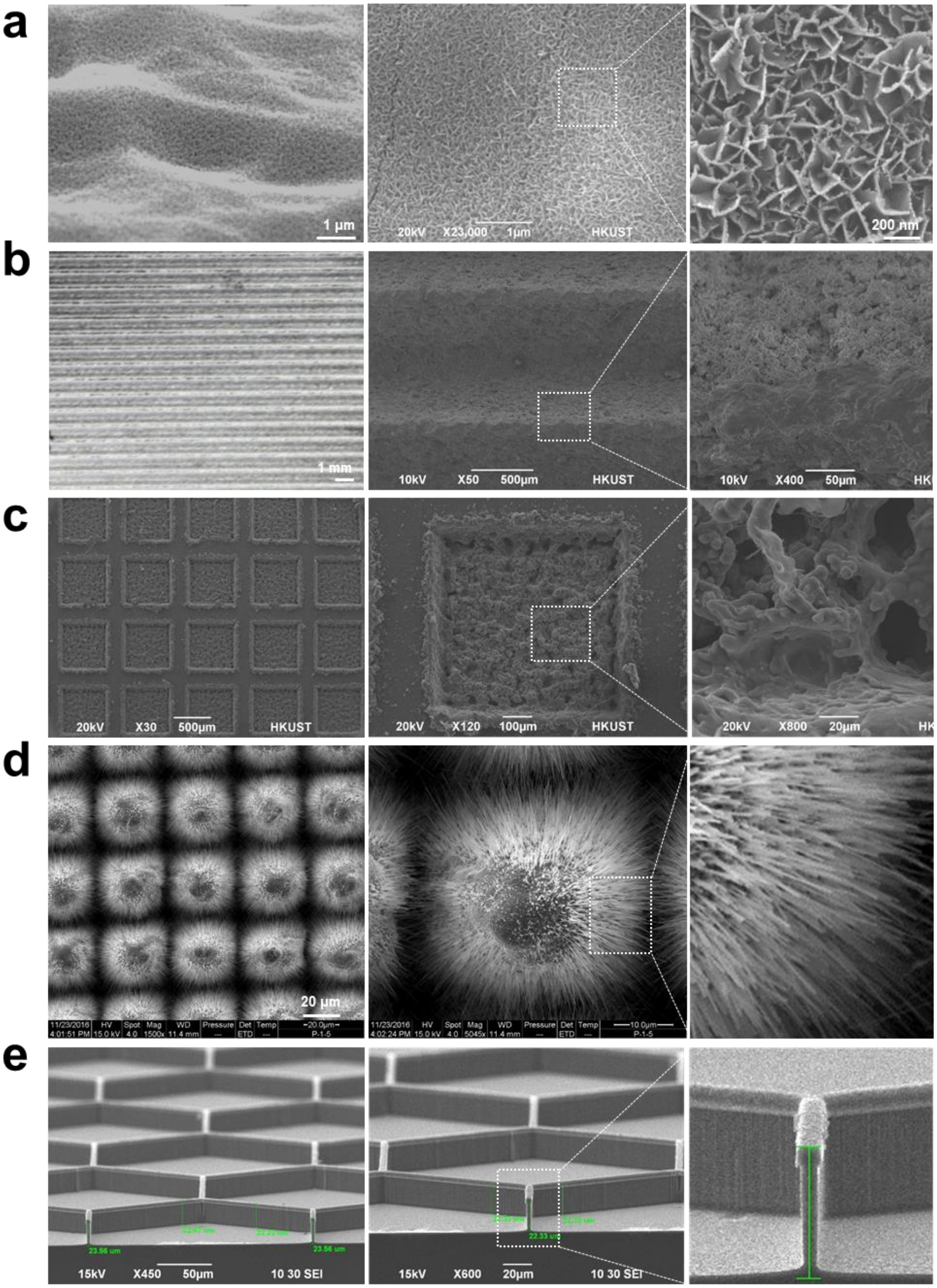

Supplementary Fig. 4. SEM of surfaces with differing surface structures used for additional droplet freezing and jumping experiments. (a) Nanostructured Al surface, labeled surface Na. The nanostructures

have a characteristic size of 100-200 nm. (b) Macro-grooved Al surface, labeled surface Ma. The triangle grooves have a pitch of 1 mm and a depth of 1 mm. (c) Micro-latticed Al surface, labeled La. The spacing and depth of the square lattices are 700 μm and ≈100 μm, respectively. (d) Hierarchical CuO nanowire surface, labeled Hi, consisting of microhills covered by CuO nanowires. The pitch and height of the microhills are 40 μm and ≈60 μm, respectively. The CuO nanowires have a diameter of ≈100 nm. (e) Re-entrant Si surface, labeled Re. The side-to-side width and height of the hexagon lattices are 200 μm and ≈23 μm, respectively. The wall of the lattice has a T-shape with a width of ≈5 μm at the top and ≈3 μm at the bottom. See Supplementary Table 1 for the contact angles of the surfaces.

Supplementary Table 1. Apparent advancing contact angle ($\theta_{\mathrm{a}}^{\mathrm{app}}$) and apparent receding contact angle ($\theta_{\mathrm{r}}^{\mathrm{app}}$) of the superhydrophobic surfaces. Samples S1-S3 are characterized in the manuscript.

Surfaces	$\theta_{\mathrm{a}}^{\mathrm{app}}$ (°)	$\theta_{\mathrm{r}}^{\mathrm{app}}$ (°)
Nanostructured Al (Na)	163±1.1	160±1.5
Macrogrooved Al (Ma)	158±1.8	155±2.2
Microlatticed Al (La)	161±1.6	159±1.8
Hierarchical CuO (Hi)	163±1.5	158±2.4
Re-entrant Si (Re)	161±2.0	144±4.5

Supplementary Notes

Section S3. Calculation of the Vaporization Flux

The vaporization rate can be estimated with known droplet surface temperature, surrounding pressure, and surrounding temperature. Using the calculated droplet surface temperature as demonstrated in Section S1, we calculated the saturation vapor pressure on both the ice surface and the supercooled water surface. The empirical relation of supercooled water vapor pressure (P_w in Pa) as a function of temperature (T in K) is given by:[12]`

$$P_\mathrm{w} = \exp\Big[-100.79 - \frac{3256.72}{T} + 24.15 \cdot \ln(T) - 0.0598 \cdot T + \tanh(T - 228.9) \cdot \Big(-21.48 + \frac{691.84}{T} + 3.54 \cdot \ln(T) - 0.00344 \cdot T\Big)\Big]. \tag{5}$$

The vapor pressure (P_i in Pa) of the ice surface as a function of surface temperature (T in ºC) is given by:[13]

$$P_\mathrm{i} = 611.21 \exp\left(\frac{22.587 \cdot T}{T + 273.86}\right). \tag{6}$$

With the calculated vapor pressure at the droplet surface ($P_\mathrm{d} = P_\mathrm{w}$ or P_i) and measured surrounding/chamber pressure P_e and temperature T_e, the net evaporation mass flux can be estimated by the Hertz-Knudsen-Schrage formula:[14]

$$J = \frac{2\beta}{2-\beta}\sqrt{\frac{M}{2\pi R}}\left(\frac{P_\mathrm{d}}{\sqrt{T_\mathrm{d}}} - \frac{P_\mathrm{e}}{\sqrt{T_\mathrm{e}}}\right), \tag{7}$$

where M is the molar mass of water vapor, β is the evaporation coefficient, R is the universal gas constant and T_d is the droplet's surface temperature (in K, ice or supercooled water). For each

experiment, the environmental pressure P_e was captured immediately before freezing. The ice sublimation mass (J_i) and the water evaporation flux (J_w) are then calculated by substituting the ice surface temperature and supercooled water temperature into Eqns. S5-S6 for each captured environmental pressure P_e. With a measured evaporation flux (Supplementary Fig. 1), the evaporation coefficient β was determined to be 0.759 in our experiments, close to the reported value (0.7) for evaporation in low pressure.[15,16] We assumed a constant evaporation coefficient for all of our low-pressure vaporization experiments. The result is plotted in Supplementary Fig. 5 and we show that within the range of environmental pressure P_e in our tests, the ice sublimation flux (J_i) is ~ 10X larger than the water evaporation flux (J_w) during the freezing process, i.e., $J_i \approx 10J_w$. Note, in the calculation of vaporation flux, we neglected the effects of non-condensable gasses (NCGs) given the low and stable environmental pressure (with continuous pumping) during freezing.

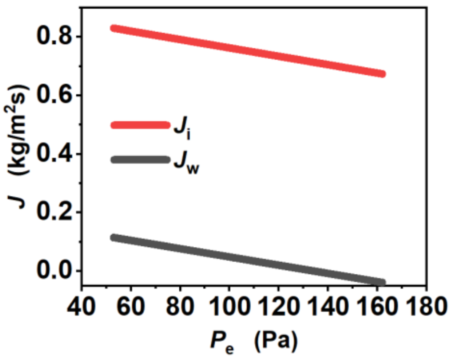

Supplementary Fig. 5. Vaporization flux of the ice surface (J_i) and supercooled liquid surface (J_w) at varying chamber pressures (P_e).

Section S4. Characterization of the Intensive Vaporization during Recalescence

Intensive vaporization is hypothesized to be the driving force for droplet jumping during recalescence. To verify the existence of such a strong vaporization flux as a result of the recalescence progression, a flow indicator (a piece of tissue string) is placed near the droplet and the chamber is depressurized as described in Supplementary Fig. 1b. Upon depressurization, the tissue string remained still, indicating the airflow of the vacuum pumping will not cause any movement of the tissue string. As the recalescence initiated at the left-hand side of the droplet and the droplet jumped to the right-hand side (indicated by the red arrow at t=7.9 ms, Supplementary Fig. 6), the tissue string accelerated to the left-hand side, which demonstrated a strong vapor flux was induced during the onset of recalescence. A similar result was also reported previously[17] where a cantilever beam fixed in one end and held near a droplet was driven to vibration upon droplet freezing under low-pressure environment. All these results provide strong evidence that the droplet freezing in low-pressure environment will induce strong vaporization flux as the recalescence propagates.

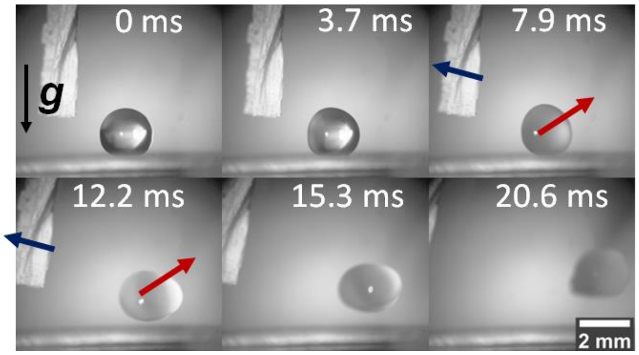

Supplementary Fig. 6. Vapor flow during vaporization. A flow indicator (tissue string) was fixed on one end while the free end was hung near the droplet. The direction of gravity is indicated. As the pressure chamber was pumped down (t<3.7 ms), the tissue remained still, indicating the pumped air flow had little effect on the string. Upon ice nucleation at t=3.7 ms, the freezing droplet jumped to the right-hand side while the tissue string accelerated to the left-hand side, demonstrating a strong vapor flux acting on the tissue string.

Section S5. Characterization of Recalescence

The droplet freezing is initiated from a nucleation site and the ice nuclei propagate rapidly over the droplet surface driven by the thermodynamic potential of the supercooled water. At the freezing moment, the local ice mass fraction within the ice-water slurry mixture can be estimated by balancing the release of latent heat and the internal energy change of the ice-water mixture, which is given by:

$$m_{\mathrm{i}} h_{\mathrm{sf}} = m_{\mathrm{i}} c_{\mathrm{i}} \Delta T + m_{\mathrm{w}} c_{\mathrm{w}} \Delta T, \tag{8}$$

$$\widetilde{m}_{\mathrm{i}} = \frac{c_{\mathrm{w}} \Delta T}{h_{\mathrm{sf}} - c_{\mathrm{i}} \Delta T + c_{\mathrm{w}} \Delta T}, \tag{9}$$

where $\widetilde{m}_{\mathrm{i}}$ is the local ice mass fraction, m_{i} is the mass of ice frozen during recalescence, m_{w} is the mass of remaining water, c_{i} and c_{w} are the specific heat of the ice and water respectively, h_{sf} is the latent heat of solidification of water (h_{sf}≈334 kJ/kg), and ΔT is the local supercooling. The energy balance (Eq. 8) has been also successfully adopted in previous studies.[18]

To predict the thickness of the ice-water slurry in the droplet, the internal temperature distribution of the droplet before freezing has to be derived. The Biot number (Bi) is estimated to determine whether a lumped capacitance model can be applied to obtain the temperature of the supercooled droplet subjected to evaporative cooling. Here, Bi is defined as the ratio of the thermal resistance of conduction to the thermal resistance of convection. $\mathrm{Bi} = h_{\mathrm{e}} L_c / k_{\mathrm{w}}$, where h_{e} is the convection heat transfer coefficient, L_{c} is the characteristic length of the droplet, and k_{w} is the thermal conductivity of water. However, the above formulation of Bi only applies to convective cooling evolved from Newton's law of cooling. To define h_{e} in terms of evaporative cooling, we plotted the actual evaporation heat flux of the droplet subjected to environmental pressure (≈100Pa) as a function of droplet temperature (Supplementary Fig. 7) based on Supplementary Eq. 11. A linear function is used to fit the actual evaporation heat flux to analogize Newton's law of cooling. The slope of the fitted line was obtained to be the fitted convection heat transfer coefficient and thus, h_{e}≈29.8 kW/m^2·K. Therefore, Bi is estimated to range from 31.8 to 97.6 depending on the droplet radius ranging from 0.6 mm to 2.0 mm. The Bi number of the droplet subjected to the evaporative

cooling is so large (Bi>>0.1) that the spatial variation of the temperature inside the droplet cannot be ignored. To calculate the time-dependent temperature field $T(r,t)$ inside the droplet as a function of time t and radial position r, a one-dimensional (1D) transient temperature modeling was applied as illustrated in Supplementary Fig. 8. The governing equation for the transient heat transfer is given in Supplementary Eq. 10, where the boundary condition (evaporative cooling) and the initial condition are shown in Supplementary Eq. 11 and Supplementary Eq. 12, respectively:

$$\frac{\partial T}{\partial t} = -\alpha\left(\frac{\partial^2 T}{\partial r^2} + \frac{2}{r}\frac{\partial T}{\partial r}\right), \tag{10}$$

$$-k_{\mathrm{w}}\frac{\partial T}{\partial r}\bigg|_{r_0} = \frac{2\beta}{2-\beta}\sqrt{\frac{M}{2\pi R}}\left(\frac{P_{\mathrm{d}}}{\sqrt{T_{\mathrm{d}}}} - \frac{P_{\mathrm{e}}}{\sqrt{T_{\mathrm{e}}}}\right)h_{\mathrm{fg}}, \tag{11}$$

$$T(r,0) = T_{\mathrm{e}}, \tag{12}$$

where α is the thermal diffusivity of water, k_{w} is the thermal conductivity of water, r_0 is the spherical radius of the droplet, β is the evaporative constant taken to be 0.759[16], M is the molar mass of water, and T_{e} is the environmental temperature (room temperature) such that we assume the initial temperature of the droplet is equal to the room temperature. P_{d} is the vapor pressure of the surface temperature of the droplet and P_{e} is the environmental pressure. A backward scheme for time discretization and a central scheme for position r discretization are chosen to solve Supplementary Equations 10 – 12.

The thickness of the ice shell and the local ice mass fraction during recalescence is local-temperature- and time-dependent. To further investigate the recalescence dynamics, we solved the temperature distribution within the droplet immediately before the onset of ice nucleation. Given the short timescale of recalescence (~30 ms) compared to the thermal conduction time scale ($\tau_{\mathrm{hc}} = L_{\mathrm{c}}^2/\alpha$) ≈ 0.824 s, where L_{c} is the radius of the droplet and α is the thermal diffusivity of water, we assumed the temperature distribution within the liquid core remains unchanged during

recalescence. To perform a transient heat transfer modeling for the droplet exposed to an evacuating chamber, we determined the transient change of humidity with the initial humidity condition (RH≈73 %, T_e=25ºC) and the water vapor capacity of the vacuum pump through the pump-down calculation equation from the data reference of the vacuum pump (Agilent DS 602 Rotary Vane Pump).[19] The estimated water vapor pressure P_v as a function of time is shown in Supplementary Fig. 9, with which we calculated the temperature distribution within the droplet over the depressurization period (t=0-17 s, Supplementary Fig. 10). Since the freezing events were happening within 15-20 s of depressurization in our experiments (Supplementary Fig. 2b), we take an average of this time scale and consider t=17 s to be the initiation of the droplet freezing in our modeling. The calculated internal temperature profile within the droplet (16 μL) at t=17 s was demonstrated in Supplementary Fig. 11a, and the local ice mass fraction immediately after recalescence, determined by Supplementary Eq. 8, was plotted as a function of position r in Supplementary Fig. 11b. The calculation shows that a significant portion of the droplet remains higher than the equilibrium freezing temperature of 273.15 K (0ºC). At t=17 s, the average thickness of the ice slurry shell reaches ≈47% of the droplet radius ($\Delta R \approx 0.47R$, Supplementary Fig. 11b), the averaged ice mass fraction within the ice-water slurry is estimated to be 10.2% (Supplementary Fig. 11b), which is adequately low such that the ice slurry shell remains a very loosen ice-water mixture and the liquid core dominates the droplet behavior during the freezing period.

The propagation velocity of recalescence can be estimated by Wilson-Frenkel equation[20] (Supplementary Eq. 13), which gives the growth velocity of ice dendrite v_g as a product of the self-diffusivity of water to ice and the thermodynamic potential difference between water and ice:

$$v_g = \frac{D(T)}{\gamma}\left[1 - \exp\left(-\frac{\Delta\mu}{k_B T}\right)\right], \tag{13}$$

where $D(T)$ is the self-diffusivity of water given in Supplementary Eq. 14,[20] γ is a constant experimentally derived to be 8×10^{-10}, and $\Delta\mu$ is the free energy difference between the water phase and ice phase given in Supplementary Eq. 15.

$$D(T) = 1.5 \times 10^{-6} \exp\left(-\frac{2045}{T}\right), \tag{14}$$

$$\Delta\mu = k_{\mathrm{B}} T \ln\left(\frac{P_{\mathrm{w}}}{P_{\mathrm{i}}}\right). \tag{15}$$

Supplementary Eq. 13 gives v_{g}≈0.089 m/s at a droplet surface temperature T=-15°C and v_{g}≈0.102 m/s at T=-20°C. The freezing time Δt_{F} during which the ice shell completely encloses the liquid core can be also estimated by Supplementary Eq. 16, which, however, may not be accurate since the calculation of v_{g} assumes isotropy in liquid water and one-dimensional ice propagation. Furthermore, v_{g} is highly supercooling dependent.

$$\Delta t_{\mathrm{F}} = \frac{\pi R_{\mathrm{d}}}{v_{\mathrm{g}}}. \tag{16}$$

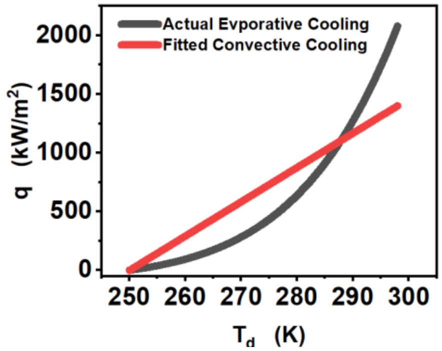

Supplementary Fig. 7. **Evaporative heat flux as a function of droplet temperature.** The black curve represents the actual evaporative heat flux calculated by Eq. 11. The gray curve is fitted by the red line which represents a fitted Newton's law of cooling in order to obtain the fitted convection coefficient h_{e}.

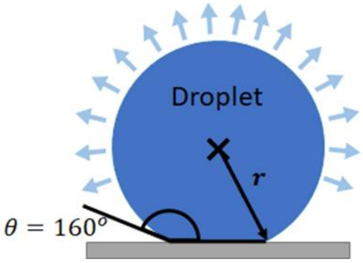

Supplementary Fig. 8. Schematic showing the transient heat transfer of a droplet subjected to evaporative cooling.

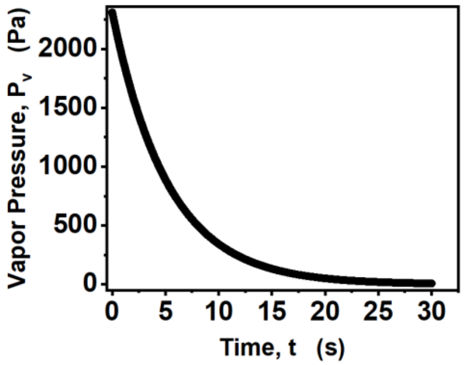

Supplementary Fig. 9. Partial pressure of water vapor as a function of time during chamber depressurization. The vapor pressure is calculated by the initial water vapor pressure within the pump and the water vapor pumping capacity. The pumping capacity is obtained from the datasheet[19] of the vacuum pump. With the water vapor pumping capacity S and the initial condition (RH≈70 %, T_e=25°C), the vapor pressure can be plotted as a function time by the pump-down equation $P_v = P_{v,0} \exp(-S \cdot t/V)$, where V is the initial volume of water vapor inside the chamber and $P_{v,0}$ is the initial water vapor pressure in the chamber.

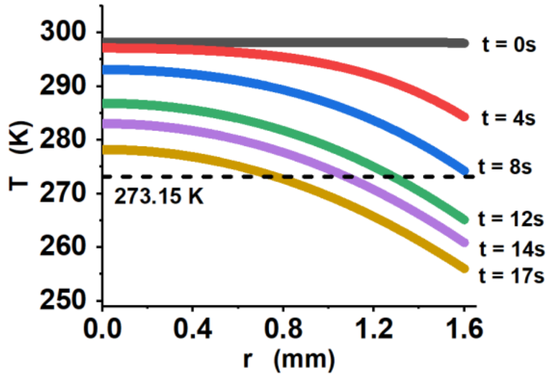

Supplementary Fig. 10. Modeled time-dependent temperature distribution within the droplet subjected to evaporative cooling. The droplet maintained a certain portion of unfrozen liquid having a temperature higher than the freezing point before t = 17 s. The modeling was performed using a time-dependent partial pressure of vapor (t = 0-17 s in Supplementary Fig. 9). Dashed line represents the equilibrium freezing temperature.

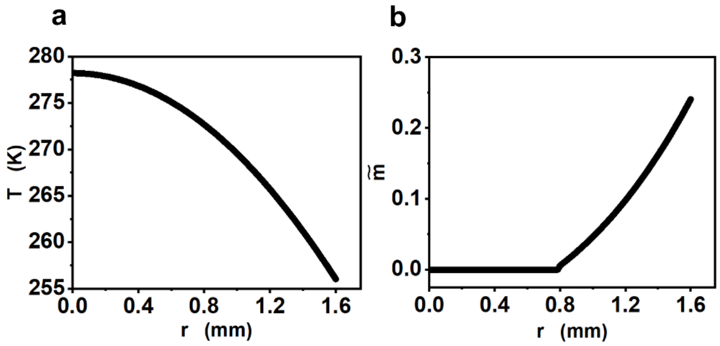

Supplementary Fig. 11. **Representative modeling results.** (a) Modeled internal temperature profile within a droplet (16 μL) immediately before ice nucleation (t = 17 s) as a function of radial position r. r=0 represents the center of the droplet sphere. (b) The local ice mass fraction as a function of radial position r at the moment when recalescence terminates.

Section S6. Vaporization-Induced Droplet Sliding

The vaporization-induced propulsion momentum results from the vaporization asymmetry of the droplet residing on a solid surface. To confirm the universality of vaporization momentum, we performed droplet depressurization-induced icing on a slippery surface with a low contact angle hysteresis (apparent advancing contact angle θ_a^{app}=100±2°, apparent receding contact angle θ_r^{app}=94±1°).[21] As shown in Supplementary Fig. 12a, the icing droplet is pushed to deform and slide on the surface as ice propagates. The droplet sliding is reflected by the traced droplet centroid shown in Supplementary Fig. 12b. Different from droplet freezing and jumping on superhydrophobic surfaces, droplet freezing on the slippery surface only enables in-plane sliding but not out-of-plane detaching due to the high droplet-surface adhesion.

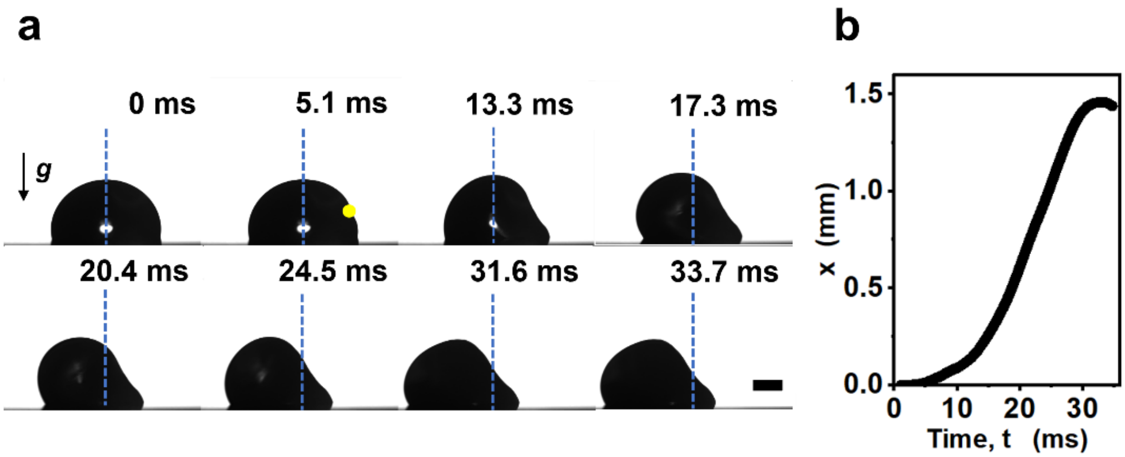

Supplementary Fig. 12. Vaporization-induced droplet sliding on a slippery surface. (a) High-speed time-lapse images of a droplet sliding on a slippery surface upon freezing during depressurization. The initial ice nucleation spot is marked by the yellow spot. The original position of the droplet is marked by the blue dashed line in each frame. Scale bar: 1 mm. (b) In-plane position of the droplet as a function of time during droplet sliding.

Section S7. Characterization of Droplet Jumping

To characterize the droplet jumping dynamics, the trajectory of the jumping droplet is captured by the high-speed camera (PCO DIMAX CS 3) at a magnification of 8X with a frame rate of up to 4588 fps. The center of mass (COM) of the droplet is determined in each frame and thus the motion of the droplet over time can be traced.[22] As demonstrated in Supplementary Fig. 13a, the COM of the droplet is moving towards the bottom left as the recalescence initiated at the up-right position of the droplet, indicating the compression of the icing droplet due to the recalescence and propulsion effect. The departure moment can be determined when the COM trajectory starts to rise after reaching the U-shape turning point (Supplementary Fig. 13a). The jumping velocity and the jumping angle at the detaching moment are determined by analyzing the COM in the X (in-plane, Supplementary Fig. 13b) and Y (out-of-plane, Supplementary Fig. 13c) direction as a function of time using the algorithm presented in previous studies.[22]

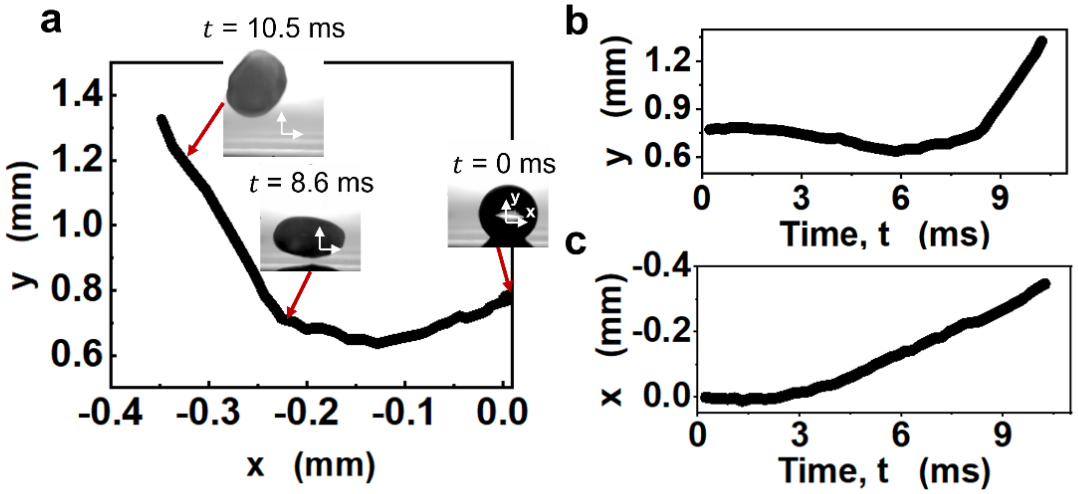

Supplementary Fig. 13. Characterization of Droplet Jumping. (a) The locus of the droplet's center of mass (COM) as the freezing droplet starts to jump from the surface. The origin is placed at the COM of the droplet immediately before droplet freezing. The jumping direction and velocity are defined at the moment when the droplet leaves the surface. Displacement in the (b) x position (horizontal) and (c) y position (vertical) as a function of time t.

Section S8. Modeling of Freezing Droplet Jumping

The jumping velocity can be modeled by assuming the detaching of the freezing droplet an elastic bouncing process,[23] where the majority of the vaporization momentum is re-directed to the droplet's bouncing momentum as the compressive liquid core elastically interacts with the superhydrophobic substrate. Considering a moment t during recalescence as seen in Supplementary Fig. 14, when the ice front reaches an angular position ϕ_{t} with respect to the center of the droplet sphere having a radius of R_{d}. Without loss of generality, the nucleation site is assumed to be at the upmost tip of the spherical droplet, where ϕ_{t}=0º at t=0 s. Given the significant contrast of vaporization fluxes on the ice surface and the supercooled water surface (J_{i}≈10J_{w}, Supplementary Fig. 5), we only account for the contribution of the vaporization at the freezing surface (J_{i}). The vaporization-induced propulsion force exerted on an arbitrary surface element within the freezing area is $dF_{\mathrm{t}} = J_{\mathrm{i}} v_{\mathrm{r}} dA_{\mathrm{r}}$, where $dA_{\mathrm{r}} = 2\pi R_{\mathrm{d}}^{2} \sin\phi d\phi$ is the area of the surface element and $\phi \in$[0º, ϕ_{t}], J_{i} is the vaporization mass flux on the ice or recalescing surface. Given the much shorter timescale of recalescence (Δt_{d}≈30 ms) compared to that of heat conduction ($\tau_{\mathrm{hc}} = L_{\mathrm{c}}^{2}/\alpha$) ≈ 0.824 s, we assume a constant temperature (T_{d}≈273.15 K or 0ºC) of the recalescing surface, which is confirmed by the IR imaging (Figure 2c in the manuscript). Thus, J_{i} can be assumed constant for a given vacuum system (Supplementary Eq. 7).[24,25] v_{r} is the vapor ejecting velocity calculated by $v_{\mathrm{r}} = J_{\mathrm{i}}/\rho_{\mathrm{v}}$.[14] The density of the water vapor near the freezing surface (273.15 K) and the supercooled water surface (≈253 K) are deduced from the ideal gas law $\rho_{\mathrm{v}} = MP/RT$. The pressure P is corresponding to saturation vapor pressure at temperature T on the icing surface (T = 273.15 K) or the supercooled water surface (T = 253 K). Therefore, we obtain the water vapor density on the icing surface to be 0.00485 kg/m^3 [26] and on the supercooled water surface to be 0.00107 kg/m^3. We note that in our experiments, v_{r} (≈144 m/s) is lower than the sonic velocity $v_{\mathrm{s}} = \sqrt{\gamma RT/M}$≈430 m/s, where γ=1.33 is the adiabatic constant for water vapor,[27] R is the universal gas constant, T is the local temperature, and M is the molar mass of water. Thus, the shock wave effects are not considered in our case.

The total net vaporization force (along the out-of-plane direction, perpendicular to the substrate) exerted on the freezing surface at time t is:

$$F_\mathrm{t} = \int_0^{\phi_\mathrm{t}} \cos\phi dF_\mathrm{t} = \int_0^{\phi_\mathrm{t}} J_\mathrm{i} v_\mathrm{r} 2\pi R_\mathrm{d}^2 \sin\phi\cos\phi d\phi = \pi R_\mathrm{d}^2 J_\mathrm{i} v_\mathrm{r} \sin^2\phi_\mathrm{t}. \tag{17}$$

The net vaporization momentum accumulated on the freezing droplet during recalescence ($t \in [0, \Delta t_\mathrm{d}]$, $\phi_\mathrm{t} \in [0, \pi]$) is:

$$\Delta p = \int_0^{\Delta t_\mathrm{d}} F_\mathrm{t} dt = \pi R_\mathrm{d}^2 J_\mathrm{i} v_\mathrm{r} \int_0^{\Delta t_\mathrm{d}} \sin^2\phi_\mathrm{t}\, dt. \tag{18}$$

where Δt_d is the recalescence/detaching time and is defined as the time counted from the ice nucleation to droplet detachment. Δt_d is found to be scaled as the inertial-capillary time[28,29] $\tau_\mathrm{c} = \sqrt{\rho_\mathrm{w} R_\mathrm{d}^3/\sigma}$ with a prefactor of 2.2 (Figure 3d of the manuscript), where σ is the surface tension of water. The prefactor is consistent with that of the characteristic timescale of an inertia/capillary-dominated oscillating droplet ($\pi/\sqrt{2}$ ≈2.2),[30] suggesting the droplet deformation and bouncing demonstrated here is likely to be dominated by the inertia and capillary forces due to the limited mass fraction of ice (≈10%, Supplementary Eq. 9). Note, the gravitational force is neglected given the Bond number Bo=0.08–0.18<1. Given a typical ice dendrite growth velocity (v_g) over the droplet surface and assuming the droplet remains a spherical shape, one finds $\phi_\mathrm{t} = v_\mathrm{g} t/R_\mathrm{d}$ and $\pi = v_\mathrm{g}\Delta t_\mathrm{d}/R_\mathrm{d}$. Thus, Supplementary Eq. 18 can be integrated:

$$\Delta p = \frac{\pi R_\mathrm{d}^2 J_\mathrm{i} v_\mathrm{r} \Delta t_\mathrm{d}}{2}. \tag{19}$$

Assuming the accumulated net momentum is eventually re-directed and transferred to the droplet's detaching momentum via the compression-bouncing dynamics of the liquid core, the jumping velocity can be obtained by:

$$\Delta p \approx m_\mathrm{d} v_\mathrm{j}, \tag{20}$$

where m_d and v_j are the mass and translational velocity of the jumping ice droplet, respectively. Given the limited mass loss due to vaporization during the short timescale of recalescence, $m_\mathrm{d} \approx \rho_\mathrm{w} V_\mathrm{d}$, where ρ_w and V_d are the density and volume of the initial water droplet, respectively. For a superhydrophobic surface with $\theta_\mathrm{a}^\mathrm{app} \approx 180^\circ$, $V_\mathrm{d} \approx \frac{4\pi}{3} R_\mathrm{d}^3$.

The droplet jumping velocity can be solved by:

$$v_\mathrm{j} \approx \frac{0.825 J_\mathrm{i}^2 R_\mathrm{d}^{0.5}}{\rho_\mathrm{v} \rho_\mathrm{w}^{0.5} \sigma^{0.5}} \sim R_\mathrm{d}^{0.5}. \tag{21}$$

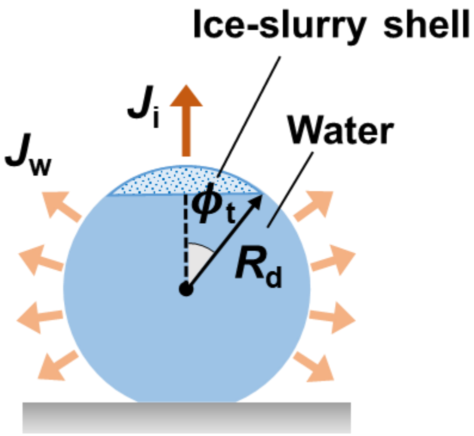

Supplementary Fig. 14. Schematics show the modeling of the vaporization momentum exerted on the freezing surface during recalescence.

Section S9. Characterization of Droplet Deformation

With the vapor propulsion force exerted on the freezing droplet during recalescence, the droplet is deformed from a spherical cap to an approximated ellipsoid. To quantify the extent of deformation, we define the deformation ratio ξ as the ratio of the surface area of the deformed droplet to that of the original droplet. The surface area of an ellipsoid is given by:[31]

$$A_{\mathrm{ep}} = 4\pi \left(\frac{a^p b^p + b^p c^p + a^p c^p}{3} \right)^{\frac{1}{p}}, \tag{22}$$

where $p \approx 1.61$ is a constant.[32] a, b and c are the major and minor axes of the ellipsoid.[20] The major axis and one of the minor axis can be determined from the side-vide high-speed video. The remaining minor axis can be obtained by assuming a constant droplet volume during deformation, i.e., the ellipsoid volume V_{ep} is equal to the initial droplet volume V_{d}, where V_{ep} is given in (Supplementary Eq. 23):

$$V_{\mathrm{ep}} = \frac{4}{3}\pi abc. \tag{23}$$

The deformation ratio ξ can be estimated by $\xi = A_{\mathrm{ep}}/A_{\mathrm{d}}$, where A_{d} is the initial droplet surface area and $A_{\mathrm{d}} \approx 4\pi R_{\mathrm{d}}^2$. Supplementary Fig. 15 shows that the droplet jumping velocity increases with the droplet deformation ratio.

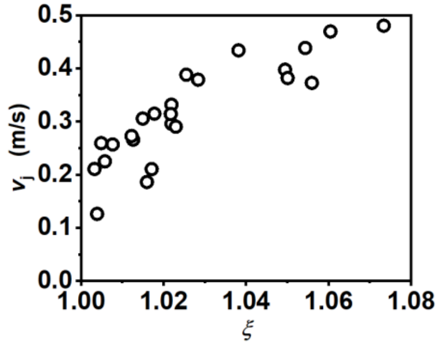

Supplementary Fig. 15. Jumping velocity v_{j} as a function of droplet deformation ratio ξ.

Section S10. Overpressure Analysis

The overpressure between the droplet base and the structured substrate has been proposed as the mechanism governing the jumping of freezing droplets.[1] To examine the effects of overpressure on droplet jumping, we derived droplet jumping velocity solely governed by the overpressure. The overpressure is estimated by:[1]

$$\Delta P_{\mathrm{op}} \approx \frac{3\mu R_{\mathrm{b}}^{2} J}{h^{3} F \rho_{\mathrm{v}}}, \qquad (24)$$

where μ is the dynamic viscosity of the vapor, R_{b} is the base radius of the droplet, J is the vaporization flux of the recalscent droplet derived from Section S3, h is the height of the microstructure, and F is the correction factor for the slip flow given as below:

$$F = 1 + \mathrm{Kn}\left(\frac{2}{\omega} - 1\right), \qquad (25)$$

where $\mathrm{Kn} = \frac{\lambda}{h}$ is Knudsen number defined by the ratio of the molecular mean free path to the height of the microstructure and ω is the tangential momentum accommodation factor,[33] which is determined to be 0.87.[1] To obtain the jumping velocity governed by the overpressure effect, the momentum conservation having a similar form to Supplementary Eq. 20 is applied, with the gravitational forces neglected:

$$\int_{0}^{\Delta t_{\mathrm{op}}} F_{\mathrm{op}} \cdot dt = \bar{F}_{\mathrm{op}} \Delta t_{\mathrm{op}} \approx m_{\mathrm{d}} v_{\mathrm{j}}, \qquad (26)$$

where F_{op} is the overpressure force exerted on the droplet base, and $\bar{F}_{\mathrm{op}}$ is the average overpressure force during the droplet-substrate contact time (Δt_{op}) starting from the development of overpressure. The droplet mass is given by:

$$m_{\mathrm{d}} = \rho_{\mathrm{w}} \frac{\pi}{3} R_{\mathrm{d}}^{3} \left(1 - \cos\theta_{\mathrm{a}}^{\mathrm{app}}\right)^{2} \left(2 + \cos\theta_{\mathrm{a}}^{\mathrm{app}}\right). \tag{27}$$

Previous experiments[1] reveal that the timescale of the development of overpressure Δt_{op} scales with the inertial-capillary time $\tau_{\mathrm{c}} = \sqrt{\rho_{\mathrm{w}} R_{\mathrm{d}}^{3} / \sigma}$:

$$\Delta t_{\mathrm{op}} = 0.32\tau_{\mathrm{c}}. \tag{28}$$

The force $\bar{F}_{\mathrm{op}}$ exerted on the droplet due to the overpressure is determined with a known droplet basal radius $R_{\mathrm{b}} = R_{\mathrm{d}} \sin\theta_{\mathrm{a}}^{\mathrm{app}}$:

$$\bar{F}_{\mathrm{op}} = \pi R_{\mathrm{b}}^{2} \Delta P_{\mathrm{op}} = \pi \left(R_{\mathrm{d}} \sin\theta_{\mathrm{a}}^{\mathrm{app}}\right)^{2} \Delta P_{\mathrm{op}}. \tag{29}$$

Thus, the jumping velocity of a droplet driven by overpressure can be solved:

$$v_{\mathrm{j}} \approx \frac{\pi\left(R_{\mathrm{d}} \sin\theta_{\mathrm{a}}^{\mathrm{app}}\right)^{2} \cdot \dfrac{3\mu\left(R_{\mathrm{d}} \sin\theta_{\mathrm{a}}^{\mathrm{app}}\right)^{2} J}{h^{3} F \rho_{\mathrm{v}}} \cdot 0.32\sqrt{\rho_{\mathrm{w}} R_{\mathrm{d}}^{3} / \sigma}}{\rho_{\mathrm{w}} \dfrac{\pi}{3} R_{\mathrm{d}}^{3} \left(1 - \cos\theta_{\mathrm{a}}^{\mathrm{app}}\right)^{2} \left(2 + \cos\theta_{\mathrm{a}}^{\mathrm{app}}\right)} \sim R_{\mathrm{d}}^{2.5}. \tag{30}$$

To validate Supplementary Eq. 30, we extracted the jumping velocity of a freezing droplet driven by overpressure reported in a previous study.[1] For the microstructured surfaces used in their studies, we obtained v_{j}=0.165 m/s, close to calculation by Supplementary Eq. 30 (v_{j} = 0.183 m/s).

To justify our hypothesis that the droplet jumping is mainly driven by vaporization momentum instead of the overpressure, the experimentally measured v_{j} is plotted in a logarithmic scale with respect to the droplet radius R_{d} as shown in Supplementary Fig. 16. The fitted slope, representing the power index, is $\approx$0.5, indicating a better interpretation of the vaporization momentum model

($v_j \sim R_d^{0.5}$) than the overpressure model ($v_j \sim R_d^{2.5}$). In addition to demonstrating the difference in the power index, we calculated the jumping velocity of the freezing droplet due to overpressure on our substrates S1-S3 through Supplementary Eq. 30, which is shown as the grey band in Figure 3e of the manuscript. The upper boundary of the band represents droplet freezing on substrate S3 with the microgroove height $h \approx 15$ μm and the lower boundary of that represents droplet freezing on substrate S2 with the microstructure height $h \approx 30$ μm. Note, due to the limited contact area between the liquid base with the microstructures (φ_m=0.223 or 0.447, or even lower given the local curvatures of the microstructures), the overpressure is assumed to mainly result from the microstructures, and the nanostructures (~100 nm) were not taken into account when calculating the overpressure using Eq. 30. The overpressure model significantly underestimates the jumping velocity, which confirms our hypothesis that the vaporization momentum dominates the freezing droplet jumping.

To further validate our model, we analyzed a recently published experimental work[34] where similar experiments were performed independently. This referenced work[34] reported the wetting transition droplet expulsion on a superhydrophobic surface under a similar low-pressure environment (~100 Pa) but did not quantify the droplet jumping kinetics. We extracted droplet jumping velocity and detachment time from their published videos (Supplementary Movies 2, 4, and 5 in Ref.34). Their experiments cover superhydrophobic surfaces having different surface patterns (micropillars and copper mesh) or varied geometries. As shown in Supplementary Fig. 17, both the extracted detachment time (Supplementary Fig. 17a) and droplet jumping velocity (Supplementary Fig. 17b) show good agreement with our model.

In the previous study[1] where the overpressure underneath the droplet is responsible for droplet jumping, the partial pressure of vapor (P_v) in the chamber during freezing, corresponding to the monitored relative humidity (<10%) and total environmental pressure (P_e~1000 Pa), is expected to be less than 300 Pa. This indicates a significant amount of non-condensable gasses (NCGs), with a partial pressure P_a=P_e-P_v>700 Pa, in the chamber during droplet vaporization. However, in our case, the environmental pressure reached an asymptotic and significantly low value of P_e~100 Pa with continued pumping before freezing, hence NCGs were limited to a minimal content. Therefore, we expect that NCGs play a negligible role in vaporization and $P_v \approx P_e$~100 Pa. Indeed, as we demonstrated in Supplementary Fig. 1d, the averaged vaporization mass flux before freezing

is 0.03 kg/m^{2}s, more than 4 times higher than the averaged vaporization flux (~ 0.0067 kg/m^{2}s) in Ref. 1. Furthermore, the determined evaporation coefficient β (0.759) in Supplementary Eq. 17 is close to the previously reported values for evaporation in pure steam conditions (β=0.7),[15,16] indicating limited effects of NCGs.

For elevated environmental pressure with increasing amount of NCGs, we quantified the effects of NCGs on vaporization by introducing the diffusion resistance R_{diff} as a function of the partial pressure of NCGs (P_{a}) and ambient temperature T_{e} as shown in Supplementary Eq. 31:[34,35]

$$R_{\mathrm{diff}} = \frac{R_{\mathrm{H_2O}} T_{\mathrm{e}} \Delta x}{0.211 \left(\frac{T_{\mathrm{e}}}{273.15}\right)^{1.94} \frac{101325}{P_{\mathrm{a}}}}, \tag{31}$$

where $R_{\mathrm{H_2O}}$ is the specific gas constant for water and Δx is the diffusion distance which we take Δx = 0.2 mm according to Ref.[34]. A higher fraction of NCGs represents a larger diffusion resistance to evaporation, leading to a reduced evaporation heat transfer coefficient. We fixed the vapor pressure (P_{v}=100 Pa) while increasing the partial pressure of NCGs (P_{a}), and calculated the vaporization mass flux J as a function of the total environment pressure ($P_{\mathrm{e}} = P_{\mathrm{v}} + P_{\mathrm{a}}$) by Supplementary Eq. 32:[34]

$$J = \frac{P_{\mathrm{d}} - P_{\mathrm{e}}}{R_{\mathrm{kin}} + R_{\mathrm{diff}}}. \tag{32}$$

Here, $R_{\mathrm{kin}} = \frac{\beta + \omega(1-\beta)}{\beta\omega}\sqrt{2\pi R_{\mathrm{H_2O}} T_{\mathrm{d}}}$ is the kinetic resistance, where T_{d} is the droplet surface temperature, P_{d} is the saturation vapor pressure at the droplet surface, β = 0.759 is the accommodation coefficient; $\omega = \frac{32\pi}{32+9\pi}$ is a constant.[34] With a known droplet radius R_{d}, the vaporization momentum and jumping velocity can be calculated by Supplementary Eq. 21. As shown in Supplementary Fig. 18, the calculated jumping velocity v_{j} induced by vaporization momentum decreases with the environmental pressure P_{e}. At $P_{\mathrm{e}} \approx 1000$ Pa, v_{j} decreases to 0.003 m/s, indicating that vaporization momentum does not contribute significantly to droplet jumping.

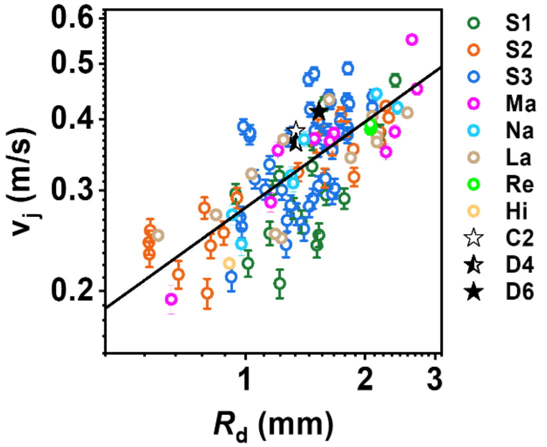

Supplementary Fig. 16. Droplet jumping velocity v_j as a function of the droplet radius R_d (in logarithmic coordinates), which is fitted by a linear function (solid line) with a slope of $\approx$0.5.

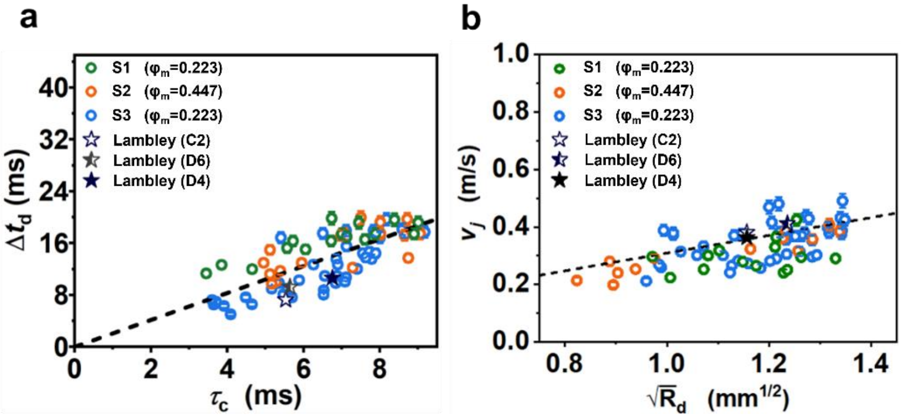

Supplementary Fig. 17. **Comparison of the literature data with our model and experiments.** (a) detachment time Δt_d as a function of inertial-capillary time τ_c. (b) Droplet jumping velocity v_j as a function of the square root of droplet radius $R_\mathrm{d}^{0.5}$. Surfaces S1, S2, and S3 having differing solid fractions with respect to microstructures (φ_m) represent experimental data in our current study. Star symbols represent data from Ref. 34 (*Lambley, H. et al. Nat. Phys., 2023*), with C2, D6, and D4 representing the copper mesh surface having a grid size of 400 μm, pillared surface having pillar spacing/diameter/height (s/d/h) of 120/10/40 μm, and pillared surface having pillar spacing/diameter/height (s/d/h) of 70/10/40 μm, respectively.

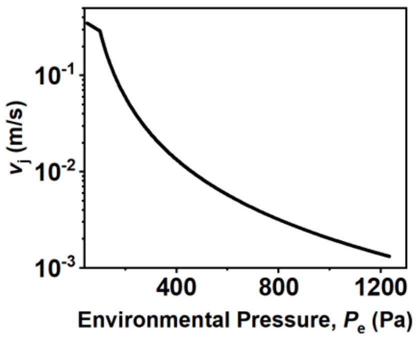

Supplementary Fig. 18. **Modeled jumping velocity v_j induced by vaporization momentum as a function of total environmental pressure P_e during freezing.** Modeling parameters: a fixed partial pressure of vapor P_v=100 Pa, droplet radius R_d=1 mm, evaporation coefficient β=0.759.

Section S11. A Qualitative Analysis of Time Scales

1) Relationship between detachment time and capillary time

We have introduced the detachment time Δt_{d} to characterize the time scale during which the droplet effectively interacts with the substrate to accumulate and redirect the vaporization momentum. Δt_{d} is defined as the time duration starting from ice nucleation to droplet-substrate separation.

To understand Δt_{d} in comparison with the timescale of other extensively studied droplet dynamics (free oscillation and bouncing), we compared Δt_{d} with the capillary time τ_{c}, and found a linear scaling ($\Delta t_{\mathrm{d}}/\tau_{\mathrm{c}} \approx 2.2$, or $\Delta t_{\mathrm{d}} \sim R_{\mathrm{d}}^{1.5}$) for the tested droplet sizes ($0.6 \leq R_{\mathrm{d}} \leq 2.8$ mm, see Supplementary Fig. 19) and experimental conditions (P_{e}~100 Pa), regardless of the variation of the tested structure geometries and length scales (see Supplementary Fig. 4). Here, $\tau_{\mathrm{c}} = \sqrt{\rho_{\mathrm{w}} R_{\mathrm{d}}^3/\sigma}$, ρ_{w} is the water density, and σ is the surface tension of water. The scaling of Δt_{d} ($\Delta t_{\mathrm{d}}/\tau_{\mathrm{c}} \approx 2.2$) is close to the characteristic timescale of a freely oscillating droplet ($\tau_{\mathrm{c}} \approx 2.2\tau_{\mathrm{c}}$) and is slightly lower than the contact time of a low-deformation impact droplet on superhydrophobic surfaces ($\tau_{\mathrm{c}} \approx 2.6\tau_{\mathrm{c}}$). [23,30] This comparison was motivated by the experimental observation that the freezing droplet experiences a compressive deformation under the recalescence-induced propulsion force while the bottom of the droplet remains unfrozen before jumping, reminiscent of the process of droplet impact and bouncing on superhydrophobic surfaces. We note that the droplet freezing, deforming, and jumping dynamics are different from the classic droplet impact and bouncing dynamics, where a droplet experiences spreading and retraction before bouncing under the capillary and inertia forces. Whereas in the freezing droplet case, the droplet deforms as the ice shell builds up, and the retraction of the liquid core is partially restricted by the ice-slurry shell. Nevertheless, the similar time scales indicate that the hydrodynamics of the liquid core within the droplet may play an important role in droplet deformation and jumping.

We assumed that during the short-time droplet deformation, the ice shell remains a thin layer of ice-water slurry mixture with a large fraction being liquid water. This assumption was justified by our calculations of the thickness and water mass fraction of the ice-water slurry shell at the detaching moment (see Supplementary Fig. 11b), showing that for a 16 µL water droplet around

the detachment moment (t=17 s), the average thickness of the ice slurry shell reaches ≈47% of the droplet radius, and the averaged ice mass fraction within the ice-water slurry is <10.2%, which is adequately low such that the ice slurry shell remains a very loosen ice-water mixture. To some extent, the hydrodynamics of ice-shelled droplets in the early stage of freezing are similar to those of particle-laden droplets or droplet marbles, which behave as a liquid when impacting solid surfaces at a low particle concentration.[36] To further confirm the assumption, we have performed additional experiments to capture the freezing dynamics at a higher capture rate (up to 20000 fps) using a high-speed camera (Photron Fastcam Nova S12) with a 12X micro lens. The enhanced temporal resolution allowed us to clearly differentiate the ice and water phases and capture the interaction between the liquid portion with the substrate (Supplementary Fig. 20). It was seen that before detaching, the liquid part of the droplet was pushed to deform and then contracted when interacting with the substate ($t \in$[8.2 ms, 12.4 ms], Supplementary Fig. 20, Supplementary Movie 3). Even at the detaching moment, part of the liquid bottom remains unfrozen (t<14.4 ms, Supplementary Fig. 20), as further reflected by the slight oscillation and contraction of the liquid after detaching (t>14.4 ms, Supplementary Fig. 20). This indicates the hydrodynamics of the droplet might be only partially confined by the ice shell during the short-time deformation process.

As we have highlighted in the manuscript, we must note that the fluid dynamics during freezing recalescence are rather complicated involving the dynamic confinement of supercooled liquid by the fast-spreading ice shell. A more fundamental study is needed to rigorously verify the relationship between detachment time and capillary time by incorporating the crystallization kinetics, ice propagation, and internal flow within the liquid core. In our current work, the demonstrated scaling of detachment time indicates that the droplet hydrodynamics at least partially govern the compressive deformation and detaching dynamics.

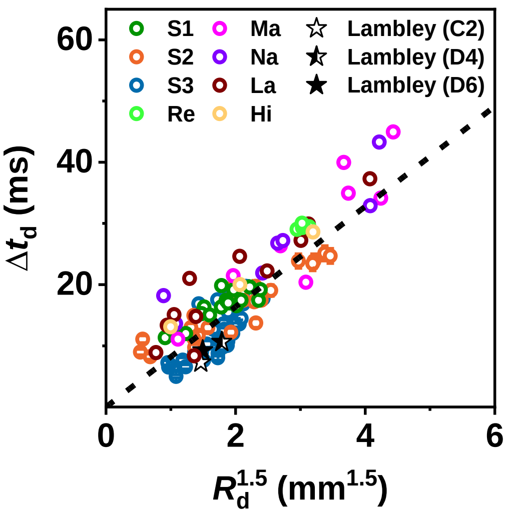

Supplementary Fig. 19. Droplet detachment time Δt_d as a function of the power of droplet radius $R_d^{1.5}$. Surfaces with differing structures (samples S1, S2, S3, Re, Ma, Na, La, Hi) were used as the substrates. Independent experimental data from the literature (*Lambley, H. et al. Nat. Phys., 2023*)[34] which used different structured surfaces (samples C2, D4, D6, star symbols) also follow the same scaling law. The dashed line represents a linear fitting. See Supplementary Fig. 4 and Table 1 for the detailed characterization of the surfaces.

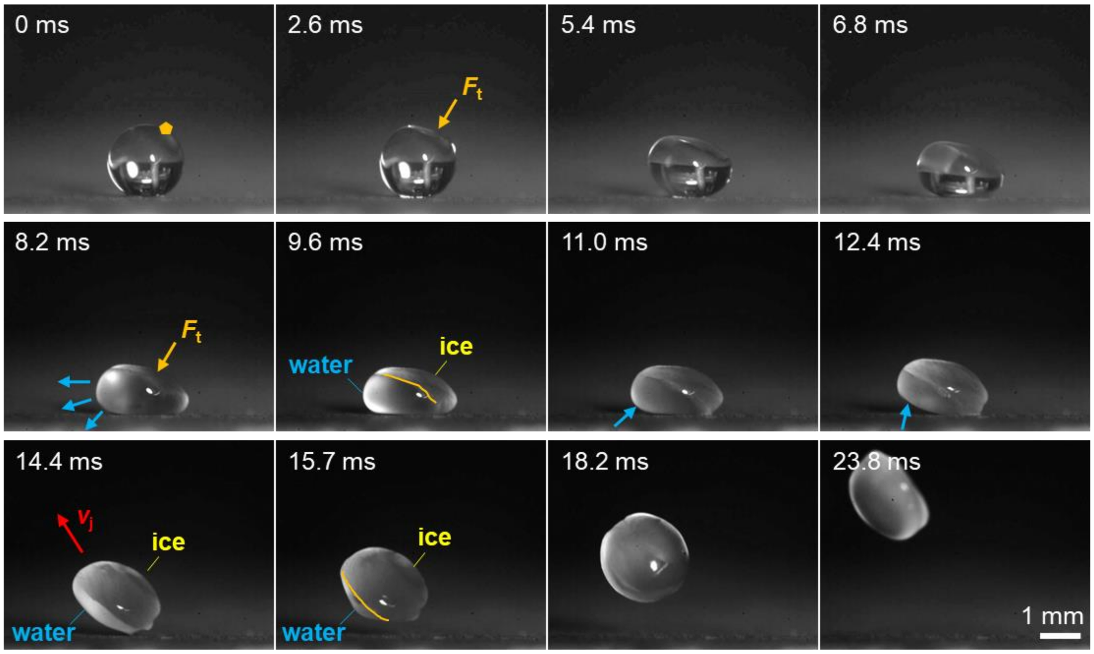

Supplementary Fig. 20. High-speed optical imaging of the droplet freezing, deforming, and self-jumping dynamics. The enhanced temporal image sequences captured the dynamics of both ice and water phases and the interaction between the liquid portion with the substrate. The initiation of ice nucleation was marked by the yellow diamond, the vaporization repulsion force (F_t) acting on the ice shell was indicated by the yellow arrow, the ice front was traced by yellow curves, the liquid spreading and retraction during

droplet compression was marked by blue arrows, and the jumping velocity (v_j) was marked by the red arrow. A superhydrophobic hierarchical CuO nanowire surface with 40-μm-spaced microhills[22] (surface Hi, see Supplementary Fig. 4) was used as the substrate. See Supplementary Movie 3.

2) Relationship between freezing time and detachment time

We have observed that the freezing time Δt_f, which represents the time needed for the ice crystal to spread over the whole water surface area, is close to (or slightly higher than) Δt_d in our experiments. This was supported by the IR and optical time-lapse images shown in Figures 2a and 2c of the manuscript as well as Supplementary Fig. 20, where we show that the ice front largely encloses the droplet at the detachment moment, with a small portion at the droplet bottom remaining unfrozen upon jumping.

Δt_f can be alternatively estimated by the crystallization kinetics, as we have demonstrated in Supplementary Eq. 16, $\Delta t_f = \pi R_d / v_g$, where v_g is the growth velocity of ice dendrite depending on the supercooling.[37] However, this calculation may only represent the lower boundary since the calculation of v_g assumes isotropy in liquid water and one-dimensional ice propagation. Furthermore, v_g is highly dependent on supercooling. In real conditions, the compressive deformation and the resultant internal flow within the liquid core enhance convective heat transfer, and the ice crystallization does not necessarily spread uniformly over the surface (see the irregular shape of the ice shell defined by the ice front, Figure 2c in the manuscript). Consequently, the freezing time cannot be well captured by Supplementary Eq. 16.

The comparable time scale of ice spreading (with a time scale of Δt_f) and compressive deformation (or detachment, with a time scale of Δt_d) of the droplet is likely due to the synchronized freezing and deforming processes. As ice nucleation initiates on the droplet surface, a propulsion force due to intensive vaporization is exerted onto the freezing surface. The net vapor propulsion force can be modeled by $F_t = \pi R_d^2 J_i v_r \sin^2 \phi$ as we have elucidated in Supplementary Eq. 17 of the Supplementary Information, where ϕ is the angular position of the freezing front with respect to the initial nucleation site. As seen in Supplementary Fig. 21a, F_t first increases as the ice spreads (ϕ<90°), reaching a maximum when half of the droplet surface is covered by ice (ϕ=90), and then decreases as the ice proceeds to enclose the droplet (ϕ>90°). Corresponding to the development of net propulsion force applied to the droplet, the droplet experiences fast compressive deformation

in the beginning until reaching the maximum deformation, and then the liquid portion is partially relaxed to allow for capillary-inertia processes as the net propulsion force starts to release (ϕ>90°, Supplementary Fig. 21b, corresponding to the freezing dynamics in Supplementary Fig. 20). Thus, the crystallization development determines the evolution of the applied net propulsion force, thus the deforming dynamics.

In our current study, we quantify the jumping velocity at the detaching moment (note that the velocity may change after detachment due to air drag, gravity, and asymmetry vaporization), and it is during the detachment time that the substrate interacts with the droplet to re-direct the vaporization momentum for out-of-plane jumping. To simplify the modeling, we assume that the recalescence completes upon detachment given the comparable time scales of freezing and detachment. Therefore, in our modeling of the vaporization momentum (Eq. 1 of the manuscript), we used the detachment time to characterize the timescale for momentum transport.

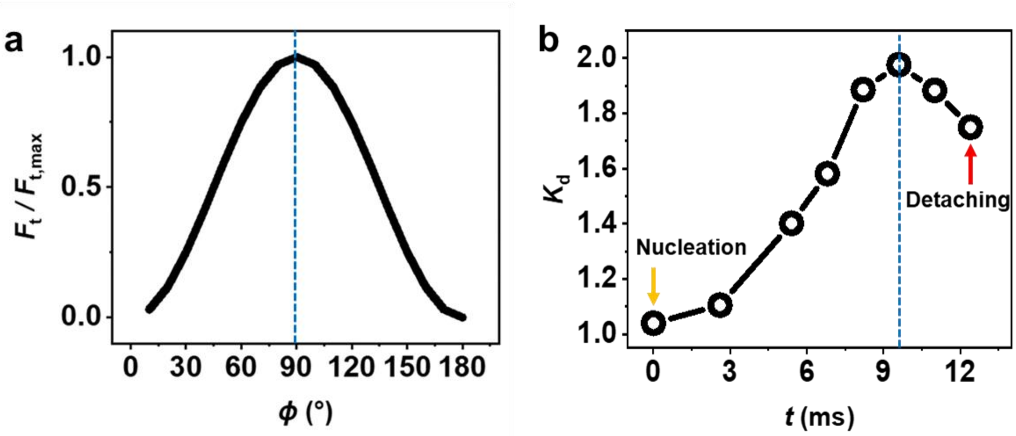

Supplementary Fig. 21. Vaporization force and droplet deformation during recalescence. (a) Calculated net vaporization repulsion force F_{t} as a function of the angular position of the ice front ϕ. A spherical droplet is assumed in the calculation. The maximum vaporization repulsion force is reached at ϕ=90° , as marked by the dashed blue line. (b) Droplet deformation, characterized by the aspect ratio (width to height) of the droplet K_{d}, as a function of time t. The nucleation and detaching moments are marked by arrows. The maximum deformation is reached before detachment, as marked by the dashed blue line.

Section S12. Effects of Substrate Adhesion on Droplet Jumping Dynamics

The substrate adhesion can resist droplet jumping from the surfaces. In our experiments, the superhydrophobic surfaces used for experiments have excellent water repellency (θ_a^{app}≈160°, θ_r^{app}>150°), thus allowing us to quantify the vaporization momentum without involving substrate adhesion.

To broaden the range of substrate wettability, we have performed additional experiments using a re-entrant Si surface with a lower level of hydrophobicity.[11] The re-entrant surface is selected because it has a high apparent advancing contact angle (θ_a^{app}≈160°) with a low apparent receding contact angle (θ_r^{app}≈140°). As shown in Supplementary Fig. 22, similar freezing, deformation, and jumping dynamics were observed on the re-entrant surface, though the captured pinning and stretching of the liquid filament at the detachment moment suggested local adhesion to the surface structures. For an identical initial droplet size (R_d=2.06 mm), the measured jumping velocity (v_j=0.39 m/s) was slightly lower (<7%) than that of a freezing droplet on superhydrophobic surfaces (v_j=0.42 m/s, obtained from the scaling relationship).

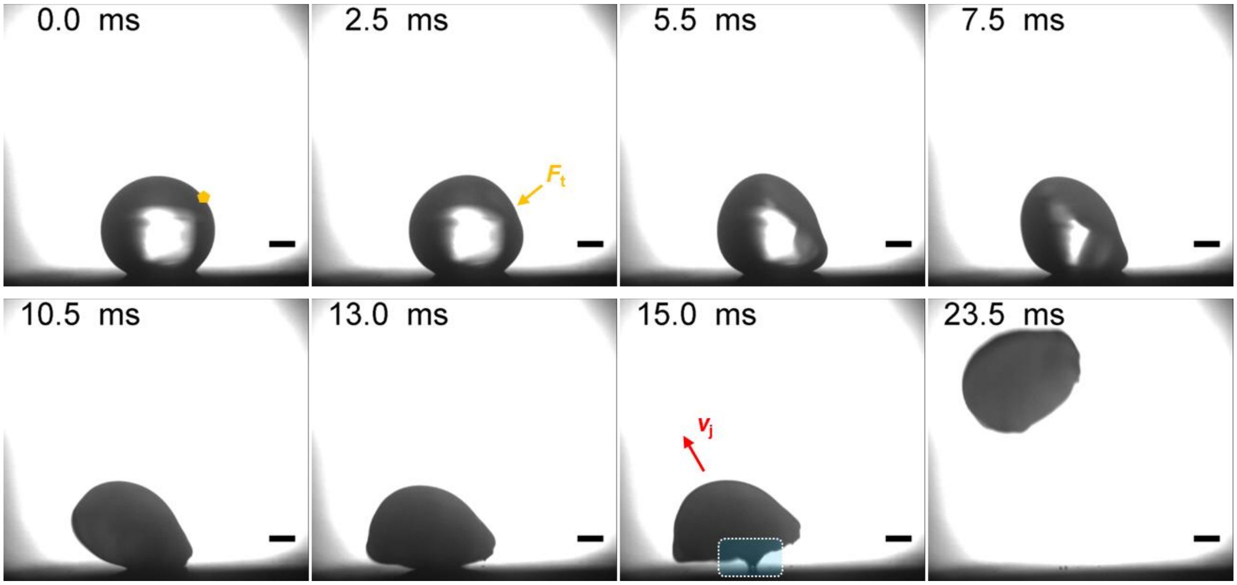

Supplementary Fig. 22. **High-speed optical imaging of droplet freezing and self-jumping on the re-entrant surface** (sample Re, see Supplementary Fig. 4 and Table 1 for detailed surface characterization). The initiation of ice nucleation was marked by the yellow diamond, the vaporization repulsion force (F_t)

acting on the ice shell was indicated by the yellow arrow, and the jumping velocity (v_j) was marked by the red arrow. The pinning and stretching of the liquid filament at the detachment moment is marked in the dotted frame. Scale bar: 1 mm.

To quantify the effects of surface adhesion on droplet jumping, we estimated the work of adhesion via $W_\mathrm{a} = \sigma(1 + \cos\theta)A_\mathrm{b}$, where A_b is the basal area of the droplet and θ is the Young's contact angle of the surface. By taking $A_\mathrm{b}= \pi R_\mathrm{b}^2$ without considering the solid fraction of the surface and θ to be the receding contact angle $\theta_\mathrm{r}^\mathrm{app}$, one can estimate the maximum work of adhesion. Here, R_b=$R_\mathrm{d}\sin\theta_\mathrm{a}^\mathrm{app}$ is the radius of the droplet's basal area. Compared with the kinetic energy of the detaching droplet, i.e., $E_\mathrm{k} = 0.5\rho V_\mathrm{d} v_\mathrm{j}^2$, where ρ is the liquid density and V_d is the volume of the jumping droplet, the work of adhesion for the surfaces and the range of droplet sizes in our experiments is relatively small ($W_\mathrm{a}/E_\mathrm{k}$<5%). Thus, we did not include the adhesion in the scaling analysis for our experiments.

The local high-energy spots or defects could introduce pinning forces to the droplet, and thus can alter the jumping direction through the pivot effects.[22] To demonstrate the effects of pinning, we introduced an artificial defect (with a length of ~500 μm and a width of ~50 μm) on the superhydrophobic substrate using a sharp knife tip (with a width of ~50 μm), deposited a droplet to the defect, and performed low-pressure freezing experiments. It was shown in Supplementary Fig. 23 that the freezing droplet detached obliquely from the surface, and the jumping direction was not correlated to the nucleation site angular position (e.g., $\theta_\mathrm{n}\approx$-56° and $\theta_\mathrm{j}\approx$-53°). This is similar to the case of coalescence induced droplet jumping on superhydrophobic surfaces with high-surface-energy pinning sites,[22] where the local pinning causes a deviation in the jumping direction, shifting it from an out-of-plane trajectory to an in-plane one. Furthermore, the jumping velocity of the pinned droplet was reduced to 0.14 m/s, in contrast to 0.24 m/s calculated from the velocity scaling for an identically sized droplet on a pinning-free surface. In our current study, we eliminated the effects of local pinning to focus on the vaporization momentum-dominated droplet jumping, and the surfaces used for the experiments all had low adhesion and uniform wettability/structures.

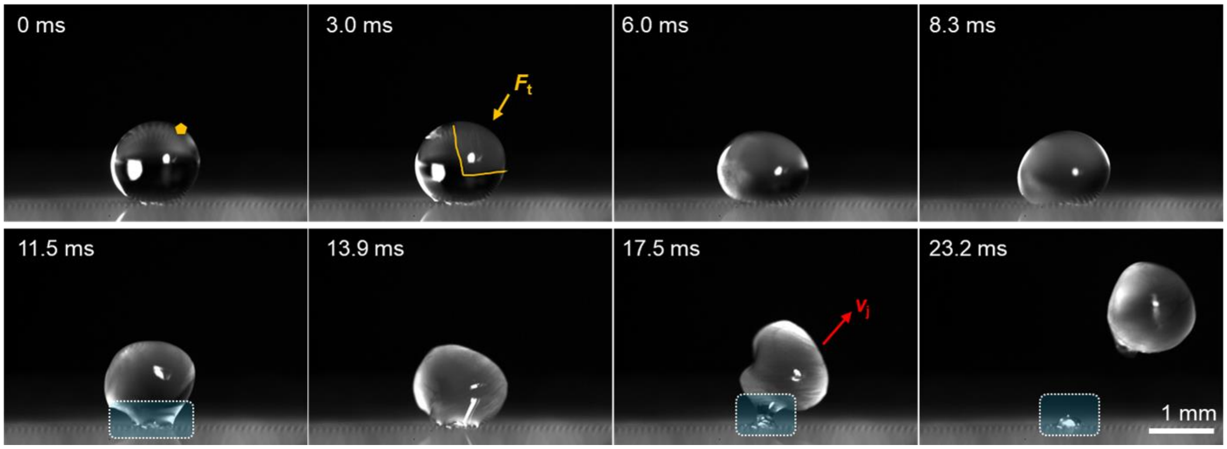

Supplementary Fig. 23. **High-speed optical imaging of droplet freezing and self-jumping on a superhydrophobic surface with a local pinning site.** High-speed optical imaging of droplet freezing and self-jumping on the micro-pillared surface (sample S1, see Figure 3c of the manuscript). The initiation of ice nucleation was marked by the yellow diamond, the vaporization repulsion force (F_{t}) acting on the ice shell was indicated by the yellow arrow, the ice front was marked by the yellow curve, and the jumping velocity (v_{j}) was marked by the red arrow. The pinning, stretching, and breakup of the liquid filament during droplet detachment are marked in the blue frame. A residual liquid filament is highlighted by the blue frame after droplet detachment.

Section S13. Development of the Regime Map

To further differentiate the overpressure and vaporization momentum, we have significantly extended the range of droplet sizes (initial R_d ranging from 0.2 to 3 mm, equivalent to a volume ranging from 0.0335 to 115 μL) in our experiments and developed a regime map (Figure 5 of the manuscript) to identify differing regimes including: (1) evaporative drying for small droplet sizes, (2) vaporization-momentum dominated droplet freezing and self-jumping for medium droplet sizes, and (3) Leidenfrost effects for large droplet sizes (Figure 5a of the manuscript). Specifically, there exists a lower boundary $R_\mathrm{d,l}$ and an upper boundary $R_\mathrm{d,u}$ in terms of droplet radius for the droplet freezing and self-jumping regime. For $R_\mathrm{d}<R_\mathrm{d,l}$, the droplet stays on the surface and dries up due to evaporation before freezing (Figures 5b and 5c of the manuscript), while for $R_\mathrm{d}>R_\mathrm{d,u}$, the droplet experiences a marginal elevation (but not jumping) by a vapor layer underneath the droplet and remarkable fluctuations at the interface (Figures 5d and 5e of the manuscript), characteristic of the Leidenfrost effect. [38,39] The freezing is largely delayed by the Leidenfrost effect due to the remarkable fluctuations of the droplet that enhance fluid convection and counteract the decrease of interface temperature.

To obtain the lower boundary $R_\mathrm{d,l}$ that separates the evaporative drying and self-jumping regimes, we compared the lifespan t_eva and the incubation time for ice nucleation t_inc of an evaporating droplet during pumping down. For a given initial droplet radius, evaporative drying occurs when $t_\mathrm{eva}<t_\mathrm{inc}$. t_eva can be obtained by considering the mass conservation during evaporation. For droplet evaporation in a depinning mode with a constant contact angle θ, which is approximately the case for our superhydrophobic surfaces having a high receding contact angle, the decrease in the droplet volume results from the vaporization over the droplet surface,[40,41] and thus:

$$\rho dV_\mathrm{d} = -JA_\mathrm{d}dt, \tag{33}$$

where $V_\mathrm{d}=\pi R_\mathrm{d}^3(1-\cos\theta)^2(2+\cos\theta)/3$ is the droplet volume as a sphere cap, $A_\mathrm{d}=2\pi R_\mathrm{d}^2(1-\cos\theta)$ is the area of the droplet's free surface, and $J=\frac{2\beta}{2-\beta}\sqrt{\frac{M}{2\pi R}}\left(\frac{P_\mathrm{d}}{\sqrt{T_\mathrm{d}}}-\frac{P_\mathrm{e}}{\sqrt{T_\mathrm{e}}}\right)$ is the vaporization mass flux given by the Hertz-Knudsen-Schrage equation [14,42] (Eq. S7, Supplementary Information) for kinetic-dominated evaporation. Integrating Supplementary Eq. 33 with a known initial droplet radius R_d gives:

$$\int_{0}^{t_{\text{eva}}} J \mathrm{d}t = \frac{\rho(1-\cos\theta)(2+\cos\theta)R_{\text{d}}}{2}, \tag{34}$$

Note, J is a function of the vapor pressure at the droplet surface (P_{d}) and vapor pressure in the environment (P_{e}), all of which are time dependent. Assuming a time-averaged vaporization flux $\bar{J}$, the droplet lifetime can be solved:

$$t_{\text{eva}} = \frac{\rho R_{\text{d}}(1-\cos\theta)(2+\cos\theta)}{2\bar{J}}. \tag{35}$$

To estimate $\bar{J}$, the transient temperature field within the droplet was simulated via COMSOL 6.0 (Heat Transfer in Fluid and Laminar Flow Physics) incorporating evaporative cooling, achieved by applying a temperature-dependent vaporization heat flux to the droplet's free surface. The modeling exploited an axisymmetric 2D geometry setup as shown in Supplementary Fig. 24a. A substrate temperature T_{sub}=298.15 K in equilibrium with the environmental temperature T_{e} was assumed. The transient environmental pressure P_{e} (Supplementary Fig. 9, Supplementary Information) was inputted as the boundary condition. Simulations were performed for an overall simulation time of 30 s with a time step of 0.1 s. This simulation time covers the time window for our depressurization experiments (Supplementary Fig. 1b of the manuscript). Droplets with varied radius R_{d}=0.2–3 mm and a constant contact angle θ=160° were simulated. Supplementary Fig. 24b presents the simulated temperature profile within a typically sized droplet, showing $T_{\text{d}}\approx$254.1 K ($P_{\text{d}}\approx$112 Pa) at t=30 s after the initiation of evaporative cooling. Simulations for differing droplet sizes (R_{d}=0.2–3 mm) showed a modest variation (2-3 K) of the temperature at the droplet's free surface at t=30 s. The simulated droplet surface temperature is a reasonable estimate when compared to the experimental measurement of the temperature inside the droplet with a similar size (-12°C, Figure 1b of the manuscript) and the derived temperature of the free surface at the freezing moment (-27°C, Supplementary Equations 1-3, Supplementary Information). $\bar{J}$ is then estimated by $\bar{J} = \frac{1}{\Delta t}\int_0^{\Delta t} J(T_{\text{d}}, P_{\text{d}}, T_{\text{e}}, P_{\text{e}})dt$ with T_{d} (and thus P_{d}) simulated as a function of time t (Supplementary Fig. 24c) and Δt approximated by 30 s. Note that an evaporation coefficient,[15,16] $\beta\approx$0.759 is adopted when evaluating J (see Section S3, Supplementary Information).

It should be noted that the simulations did not incorporate changes in droplet size during evaporation and assumed negligible effects of droplet mass depletion on the droplet temperature profile. This assumption is based on the fact that the droplet size does not change significantly during the rapid pumping-down process. The simulations were only used to estimate the droplet surface temperature for the analytical modeling (Supplementary Eq. 35) and were not performed to obtain the lifetime of evaporating droplets, which is feasible but numerically challenging to include the phase change (mass depletion) at the interface and internal circulation within the droplet. Instead of dealing with the numerical convergence issues, we used the simulations to estimate the surface temperature and to develop a semi-analytical model of the lifetime of evaporating droplets.

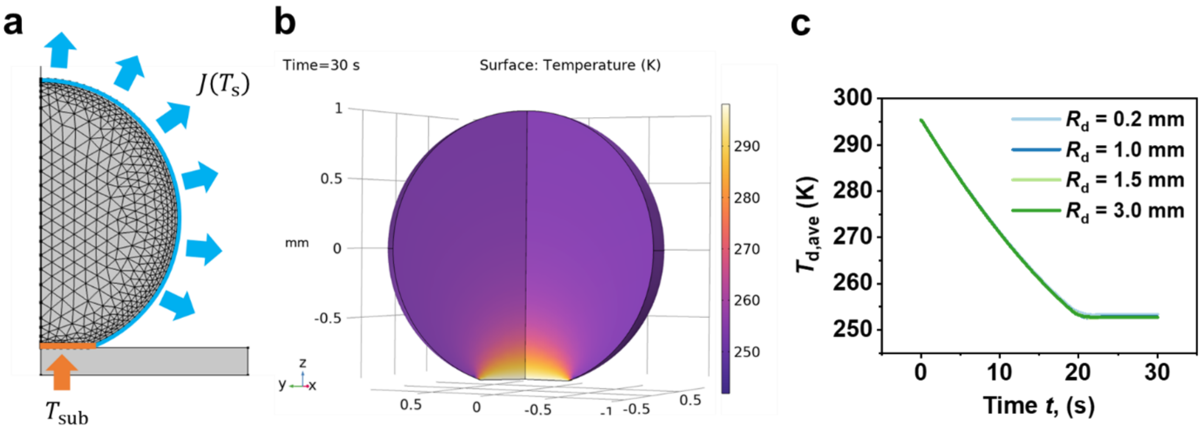

Supplementary Fig. 24. Simulated droplet temperature profile during evaporative cooling. (a) COMSOL multiphysics model setup of droplet evaporation, where the free surface of the droplet is subjected to the evaporation cooling flux $J(T_d)$ as a function of surface temperature T_d. The transient environmental pressure P_e (Supplementary Fig. 9) was inputted as the boundary condition. Simulation was performed with a contact angle θ=160° and a time step of 0.1 s. (b) Simulated temperature distribution at time t=30 s for a droplet having a radius R_d=1.5 mm. Evaporative cooling starts at t=0 s. (c) The averaged droplet surface temperature $T_{d,ave}$ as a function of time t. Minimal differences in $T_{d,ave}$ were observed for R_d ranging from 0.2 to 3 mm.

The ice nucleation incubation time t_{inc} corresponds to the moment when the very first nucleus is formed in the droplet. The total number of nuclei is a function of the droplet volume and the ice nucleation rate. The latter is dependent on the temperature field inside the droplet. Here, the transient temperature field of droplets with different sizes was obtained from the numerical

simulation performed above (Supplementary Fig. 24). With a known time-dependent temperature field, the local nucleation rate per unit volume is given by the classical nucleation theory: [37]

$$J_{\mathrm{n}} = \frac{A_{\mathrm{m}}}{3} n^{7/3} \sqrt{\frac{A_{\mathrm{m}}\sigma}{\pi k_{\mathrm{B}} T}} D_{\mathrm{w}} \exp\left\{ -\frac{4\left(\frac{A_{\mathrm{m}}\sigma}{k_{\mathrm{B}}T}\right)^3}{27\left[\ln\left(\frac{P_{\mathrm{w}}}{P_{\mathrm{i}}}\right)\right]^2} \right\}, \tag{36}$$

where $A_{\mathrm{m}} = 4\pi r_{\mathrm{m}}^2$ is the surface area of a water molecule with a radius of r_{m}, $n = \frac{\rho N}{M}$ is the number density of the water, ρ is water density, Avogadro constant N, and molar mass M, $D_{\mathrm{w}} = 1.5 \times 10^{-6} \exp\left(-\frac{2045}{T}\right)$ is the self-diffusivity of supercooled water, $\sigma = 0.0291 + 10^{-4}(T - 273.15)$ is the water-ice interfacial tension, P_{w} is the vapor pressure of supercooled water at temperature T, and P_{i} is the vapor pressure of ice at an equilibrium freezing temperature of 273.15 K. The condition of ice nucleation is defined when the total number of ice nuclei exceeds unity, given by integrating the local ice nucleation rate J_{n} over the droplet volume during a period of t_{inc}:[18,37,43]

$$\int_0^{t_{\mathrm{inc}}} \int_{V_{\mathrm{d}}}^{V_{\mathrm{d,t}}} J_{\mathrm{n}}\, dV_{\mathrm{d}} dt = 1, \tag{37}$$

where $V_{\mathrm{d,t}}$ is the droplet volume at time t and can be solved by Supplementary Eq. 33. For a given initial droplet volume V_{d}, t_{inc} can be found by numerically solving the integration problem above with the input of the time-dependent droplet temperature profile. Given that the droplet temperature is lower than the substrate (Supplementary Fig. 24), we only considered the homogeneous nucleation and neglected the heterogeneous nucleation at the droplet-substate interface. Figure 5c of the manuscript shows t_{inc} as a function of the initial droplet radius R_{d}, revealing that a smaller droplet has a longer incubation time for ice nucleation. This is because a smaller surface area or volume leads to a reduced number of ice nuclei. Moreover, the small droplet has a lower temperature that is closer to the substrate temperature due to its lower thermal conduction resistance.

By comparing the ice incubation time and the lifetime of the evaporating droplet, a critical droplet size $R_{\mathrm{d,l}}$, below which ice nucleation hardly occurs, can be solved by:

$$t_{\mathrm{eva}} < t_{\mathrm{inc}}. \tag{38}$$

The calculated $R_{\mathrm{d,l}}$ is shown to be consistent with the experiments (Figures 5a and 5c in manuscript).

To obtain the upper droplet size boundary $R_{\mathrm{d,u}}$ that separates the Leidenfrost effect and the self-jumping regimes, we consider the criteria for the initiation of the Leidenfrost effect, which starts when the overpressure force F_{op} underneath the droplet overcomes the gravity and adhesion of the droplet. Here, gravitational force (F_{g}) and adhesion force (F_{a}) are incorporated as the droplet has a significantly large size (Bo>1) and a non-spherical shape with increased droplet-surface contact area. The overpressure force F_{op}, defined consistently with that in our analyses of overpressure (Figure 3e in the manuscript and Supplementary Equations 26-29 in the Supplementary Information), can be modeled by:[1]

$$F_{\mathrm{op}} \approx \frac{3\mu\pi R_{\mathrm{b}}^4 J}{h^3 F \rho_{\mathrm{v}}}, \tag{39}$$

where μ is the dynamic viscosity of vapor, R_{b} is the basal radius of the droplet, J is the evaporation flux of the droplet, h is the height of the surface structure, $F = 1 + Kn\left(\frac{2}{\omega} + 1\right)$ is the correction factor for the slip flow with respect to the Knudsen number Kn and the tangential momentum accommodation factor ω=0.87,[1] and ρ_{v} is the vapor density corresponding to the droplet-substrate interfacial temperature.

The evaporation flux (J) can be calculated by Supplementary Eq. 7 of the Supplementary Information. Due to the absence of freezing, J is evaluated by the temperature of supercooled water at the droplet-substrate interface rather than the freezing temperature. The droplet-substrate interfacial temperature can be approximated by the average of the substrate temperature and the droplet’s free surface temperature. The latter was obtained from the numerical simulations (Supplementary Fig. 24), which demonstrated a free surface temperature of ≈253.4 K at t=30 s, with minimal dependence on the droplet radius (0.6-3 mm). We note that t=30 s is adequately long for the environmental pressure to stabilize and for the droplet to reach a relatively stable

temperature, thus the simulated droplet surface temperature could be a reasonable estimate of the temperature at the moment of droplet levitation.

The adhesion force F_a can be calculated by considering the contact angle hysteresis of the surface:[22,44,45]

$$F_a = 2\pi\sigma R_b(1 + cos\,\theta_r), \tag{40}$$

where σ is the surface tension of the water, R_b is the basal radius of the droplet at the moment of levitation, θ_r is the receding contact angle that is assumed to be close to the apparent receding contact angle θ_r^{app}.

The gravitational force of the droplet F_g is obtained by:

$$F_g = \rho V_d g, \tag{41}$$

where ρ is the density of water, g is the gravitational acceleration, V_d is the droplet volume at the levitation moment and is a function of the droplet basal radius R_b. For Bond numbers of droplets close to 1, the droplet can no longer hold a spherical cap shape. In this case, the volume of the droplet as a function of the basal radius R_b can be derived by using a polynomial to interpolate the volume-radius relationship given by Ref.46:

$$V_d\ [\mu L] = 16.164R_b^2 + 4.6477R_b + 4.7753\ [R_b \text{ in mm}], \tag{42}$$

which is interpolated for a droplet with a contact angle of 162°. A critical droplet volume V_d is then solved by Supplementary Eq. 43 below and converted to a critical droplet radius $R_{d,u}$ (of a sphere with an equivalent volume of V_d). The calculation is consistent with our experiments (Figures 5c and 5e).

$$F_{op} > F_a + F_g. \tag{43}$$

Although a large and stationary droplet necessitates a shorter timescale to incubate ice nucleation (due to a large surface area), it has a higher volume-averaged temperature as we have demonstrated in Supplementary Fig. 25 via numerical simulations (COMSOL 6.0 Heat Transfer in Fluid and Laminar Flow Physics under an axisymmetric 2D geometry). Here, the average temperature of a droplet was defined by the average of the maximum and minimum temperatures within the droplet.

Furthermore, the temperature profile within the droplet can be significantly flattened due to the remarkable fluctuations of the Leidenfrost droplet that enhances fluid convection. Therefore, freezing is delayed by the Leidenfrost effect.

Once the Leidenfrost effect is initiated via overpressure, a vapor layer is formed to provide shear pressure that elevates the droplet.[38] The Leidenfrost phenomenon is characterized by a vapor film underneath the droplet that elevates the droplet from the substrate, which shares similarities to the overpressure effects that drive droplet trampolining.[1] However, the difference between overpressure and the shear stress driving the Leidenfrost effect is that the Leidenfrost droplet detaches locally and slightly from the surfaces and has reduced dependence on surface structures, while the overpressure theory considers the situation when the droplet contacts closely with the structured surface and the limited drainage of the vapor through the structures strengthens the local pressure. Furthermore, the thickness of the vapor layer for a Leidenfrost droplet is highly correlated to the weight of the droplet, and thus the local pressure decreases rapidly with the vapor layer thickness.[38]

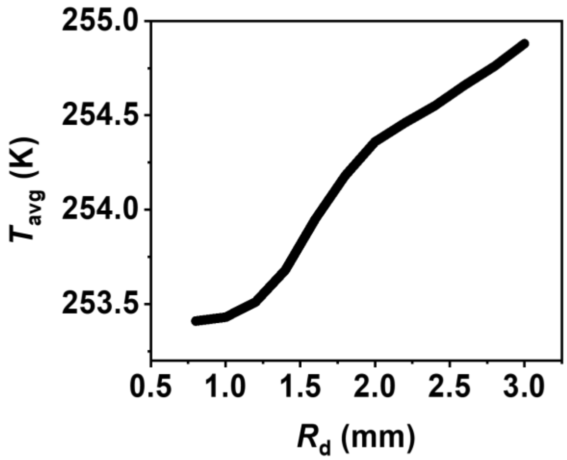

Supplementary Fig. 25. Simulated volume-averaged droplet temperature T_{avg} as a function of droplet size R_d ranging from 0.6–3 mm for a water droplet exposed to a low-pressure environment. Simulations were performed via COMSOL 6.0 (Heat Transfer in Fluid and Laminar Flow Physics) under an axisymmetric 2D geometry. The transient depressurizing pressure as a function of time (Supplementary Fig. 9), a constant substrate temperature of 298.15 K, a fixed droplet shape (θ=160°), and a no-slip boundary condition on the droplet's free surface for simplicity were applied. The simulation was carried out in a time-dependent model, where the time span of the simulation was 30 s, adequately long for the stabilization of the environmental pressure. The average temperature was extracted at 30 s.

Supplementary Reference

1. Schutzius, T. M. *et al.* Spontaneous droplet trampolining on rigid superhydrophobic surfaces. *Nature* **527**, 82–85 (2015).
2. Chandramohan, A., Weibel, J. A. & Garimella, S. V. Spatiotemporal infrared measurement of interface temperatures during water droplet evaporation on a nonwetting substrate. *Appl. Phys. Lett.* **110**, 041605 (2017).
3. Fukatani, Y. *et al.* Effect of ambient temperature and relative humidity on interfacial temperature during early stages of drop evaporation. *Phys. Rev. E* **93**, 043103 (2016).
4. Zhao, G. *et al.* Competing Effects between Condensation and Self-Removal of Water Droplets Determine Antifrosting Performance of Superhydrophobic Surfaces. *ACS Appl. Mater. Interfaces* **12**, 7805–7814 (2020).
5. He, J.-G. *et al.* Fabrication of Metallic Superhydrophobic Surfaces with Tunable Condensate Self-Removal Capability and Excellent Anti-Frosting Performance. *Nanomaterials* **12**, 3655 (2022).
6. Miljkovic, N. *et al*. Jumping-droplet-enhanced condensation on scalable superhydrophobic nanostructured surfaces. *Nano Lett.* **13**, 179-187 (2013).
7. von Rohr, P. R. *et al.* Droplet self-propulsion on superhydrophobic microtracks. *ACS Nano* **14**, 12895–12904 (2020).
8. Ma, W. *et al.* Solar-assisted icephobicity down to −60°C with superhydrophobic selective surfaces. *Cell Rep. Phys. Sci.* **2**, 100384 (2021).
9. Yan, X. *et al.* Atmosphere-mediated scalable and durable biphilicity on rationally designed structured surfaces. *Adv. Mater. Interfaces* **7**, 2000475 (2020).
10. Yan, X. *et al.* Atmosphere-mediated superhydrophobicity of rationally designed micro/nanostructured surfaces. *ACS Nano* **13**, 4160–4173 (2019).
11. Liao, D., Yang, Y. & Qiu, H. Droplet impact dynamics and heat transfer on nanostructured doubly reentrant cavity under freezing temperature. *Phys. Fluids* **33**, 052005 (2021).
12. Jana, K. Vapor pressure of supercooled water. *Int. J. Thermophys.* **43**, 165 (2022).
13. Huang, J. A simple accurate formula for calculating saturation vapor pressure of water and ice. *J. Appl. Meteorol. Climatol.* **57**, 1265–1272 (2018).
14. Gerasimov, D. N. & Yurin, E. I. *Kinetics of Evaporation*. vol. 68 (Springer International Publishing, Cham, 2018).
15. Narusawa, U. & Springer, G. S. Measurements of evaporation rates of water. *J. Colloid Interface Sci.* **50**, 392–395 (1975).
16. Eames, I. W., Marr, N. J. & Sabir, H. The evaporation coefficient of water: a review. *Int. J. Heat Mass Transf.* **40**, 2963–2973 (1997).
17. Graeber, G., Dolder, V., Schutzius, T. M. & Poulikakos, D. Cascade freezing of supercooled water droplet collectives. *ACS Nano* **12**, 11274–11281 (2018).

18. Meng, Z. & Zhang, P. Freezing dynamics of supercooled micro-sized water droplets. *Int. J. Heat Mass. Transf.* **193**, 122955 (2022).

19. Agilient. Two Stage 25 m3/h (17.6cfm) Rotary Vane Pump, DS 602. *https://www.agilent.com/en/product/vacuum-technologies/oil-sealed-rotary-vane-pumps/dual-stage-ds-rotary-vane-pumps/ds-602-rotary-vane-pump#literature*.

20. Xu, Y., Petrik, N. G., Smith, R. S., Kay, B. D. & Kimmel, G. A. Growth rate of crystalline ice and the diffusivity of supercooled water from 126 to 262 K. *Proc. Natl. Acad. Sci. U.S.A.* **113**, 14921–14925 (2016).

21. Yan, X. *et al.* Microscale Confinement and Wetting Contrast Enable Enhanced and Tunable Condensation. *ACS Nano* **16**, 9510–9522 (2022).

22. Yan, X. *et al.* Droplet jumping: effects of droplet size, surface structure, pinning, and liquid properties. *ACS Nano* **13**, 1309–1323 (2019).

23. Richard, D., Clanet, C. & Quéré, D. Contact time of a bouncing drop. *Nature* **417**, 811–811 (2002).

24. Pan, Z., Dash, S., Weibel, J. A. & Garimella, S. v. Assessment of water droplet evaporation mechanisms on hydrophobic and superhydrophobic substrates. *Langmuir* **29**, 15831–15841 (2013).

25. Nguyen, T. A. H., Biggs, S. R. & Nguyen, A. v. Analytical model for diffusive evaporation of sessile droplets coupled with interfacial cooling effect. *Langmuir* **34**, 6955–6962 (2018).

26. Yunus A. Cengel & Michael A. Boles. Property Table and Charts. in *Thermodynamics: An Engineering Approach* (McGraw Hill).

27. Frank M. White. Physical Properties of Fluids. in *Fluid Mechanics* (McGraw-Hill).

28. Yu, X., Zhang, Y., Hu, R. & Luo, X. Water droplet bouncing dynamics. *Nano Energy* **81**, 105647 (2021).

29. Liu, F., Ghigliotti, G., Feng, J. J. & Chen, C.-H. Numerical simulations of self-propelled jumping upon drop coalescence on non-wetting surfaces. *J. Fluid. Mech.* **752**, 39–65 (2014).

30. Rayleigh, Lord. On the capillary phenomena of jets. *Proc. Royal Soc. Lond.* **29**, 71–97 (1879).

31. Yan, X. *et al.* Laplace Pressure Driven Single-Droplet Jumping on Structured Surfaces. *ACS Nano* **14**, 12796–12809 (2020).

32. Lehmer, D. H. Approximations to the area of an n -dimensional ellipsoid. *Can. J. Math.* **2**, 267–282 (1950).

33. Bahrami, M., Tamayol, A. & Taheri, P. Slip-Flow Pressure Drop in Microchannels of General Cross Section. *J. Fluids Eng.* **131**, 031201 (2009).

34. Lambley, H. *et al.* Freezing-induced wetting transitions on superhydrophobic surfaces. *Nat. Phys.* **19**, 649–655 (2023).

35. Pruppacher, H. R. & Klett, J. D. *Microphysics of Clouds and Precipitation*. vol. 18 (Springer Netherlands, Dordrecht, 2010).

36. Lathia, R., Modak, C. D. & Sen, P. Two modes of contact-time reduction in the impact of particle-coated droplets on superhydrophobic surfaces. *Droplet* **2**, e89 (2023).

37. Meng, Z. & Zhang, P. Dynamic propagation of ice-water phase front in a supercooled water droplet. *Int. J. Heat Mass Transf.* **152**, 119468 (2020).

38. Shi, M., Das, R., Arunachalam, S. & Mishra, H. Suppression of Leidenfrost effect on superhydrophobic surfaces. *Phys. Fluids* **33**, 122104 (2021).

39. Yang, J. *et al.* A standing Leidenfrost drop with Sufi whirling. *Proc. Natl. Acad. Sci. U.S.A.* **120**, e2305567120 (2023).

40. Nguyen, T. A. H. & Nguyen, A. V. On the lifetime of evaporating sessile droplets. *Langmuir* **28**, 1924–1930 (2012).

41. Kang, F., Shen, Y., Cheng, Y. & Li, N. Lifetime prediction of sessile droplet evaporation with coupled fields. *Ind. Eng. Chem. Res.* **60**, 15782–15792 (2021).

42. Vaartstra, G., Lu, Z., Lienhard, J. H. & Wang, E. N. Revisiting the Schrage equation for kinetically limited evaporation and condensation. *J. Heat Transfer.* **144**, 080802 (2022).

43. Castillo, J. E., Huang, Y., Pan, Z. & Weibel, J. A. Quantifying the pathways of latent heat dissipation during droplet freezing on cooled substrates. *Int. J. Heat Mass Transf.* **164**, 120608 (2021).

44. Yan, X. *et al.* Particulate–Droplet Coalescence and Self-Transport on Superhydrophobic Surfaces. *ACS Nano* **16**, 12910–12921 (2022).

45. Konstantinidis, K., Göhl, J., Mark, A. & Sasic, S. Coalescence-induced jumping of droplets from superhydrophobic surfaces—The effect of contact-angle hysteresis. *Phys. Fluids* **34**, 113302 (2022).

46. Yu, Y., Lv, C., Wang, L. & Li, P. The Shape of heavy droplets on superhydrophobic surfaces. *ACS Omega* **5**, 26732–26737 (2020).
