# Supplementary material for: Unraveling the role of vaporization momentum in self-jumping dynamics of freezing supercooled droplets at reduced pressures
Source: Nat Commun. 2024 Feb 21;15:1567. doi: 10.1038/s41467-024-45928-2 (PMC10879204; doi:10.1038/s41467-024-45928-2)
Supplement: Supplementary file 3 — Description of Additional Supplementary Files [file 41467_2024_45928_MOESM3_ESM.docx]

**File Name:** Supplementary Movie 1

**Description:** High-speed optical imaging showing droplet freezing and jumping on a superhydrophobic surface at reduced pressure. (a) Side view. (b) Top view. Videos were captured at 8X magnification with a capture rate of 4588 fps in (a) and 2000 fps in (b). The video in (a) is played back at 450X slower and in (b) is played back at 300X slower. The droplet size in both experiments was ~ 10 μL. The droplet freezing was performed in the chamber having an environmental pressure $P_{e}$ ~ 100 Pa and environmental temperature $T_{e}$ = 25 ± 1 ^o^C.

**File Name:** Supplementary Movie 2

**Description:** High-speed infrared (IR) imaging showing droplet freezing and jumping on a superhydrophobic surface at reduced pressure. The video was captured at 4X magnification with a capture rate of 300 fps. The video is played back at 150X slower. The superhydrophobic substrate sample S3 was used and the droplet size was ~ 10 μL. The droplet freezing was performed in the chamber having an environmental pressure $P_{e}$ ~ 100 Pa and environmental temperature $T_{e}$ = 25 ± 1 ^o^C. A sapphire window was used to ensure infrared transparency.

**File Name:** Supplementary Movie 3

**Description:** High-speed optical imaging of droplet freezing and jumping on a superhydrophobic surface at reduced pressure, showing the dynamic liquid-solid interactions. The video was captured at a capture rate of 7200 fps and is played back at 10 fps. The initial droplet radius was ≈0.94 mm. The droplet freezing was performed in the chamber having an environmental pressure $P_{e}$ ~ 100 Pa and environmental temperature $T_{e}$ = 25 ± 1 ^o^C.

**File Name:** Supplementary Movie 4

**Description:** High-speed optical imaging showing evaporative drying of a small droplet at reduced pressure. The video was captured with a capture rate of 142 fps and is played back at real time speed. The initial droplet size was ~ 1 μL. The experiment was performed in the chamber having an environmental pressure $P_{e}$ ~ 100 Pa and environmental temperature $T_{e}$ = 25 ± 1 ^o^C.

**File Name:** Supplementary Movie 5

**Description:** High-speed optical imaging showing overpressure-initiated Leidenfrost effects of a large droplet at reduced pressure. The video was captured with a capture rate of 965 fps and is played back at 20X slower. The initial droplet size was ~ 100 μL. The experiment was performed in the chamber having an environmental pressure $P_{e}$ ~ 100 Pa and environmental temperature $T_{e}$ = 25 ± 1 ^o^C.
